# Myc Cooperates with Ras by Programming Inflammation and Immune Suppression

**DOI:** 10.1016/j.cell.2017.11.013

**Published:** 2017-11-30

**Authors:** Roderik M. Kortlever, Nicole M. Sodir, Catherine H. Wilson, Deborah L. Burkhart, Luca Pellegrinet, Lamorna Brown Swigart, Trevor D. Littlewood, Gerard I. Evan

**Affiliations:** 1 Department of Biochemistry, University of Cambridge, 80 Tennis Court Road, Cambridge CB2 1GA, UK; 2 Department of Pathology and Helen Diller Family Comprehensive Cancer Center, University of California, San Francisco, San Francisco, CA 94143, USA

**Keywords:** Myc, oncogene cooperation, lung cancer, tumor microenvironment, IL-23, CCL9, immune suppression, Ras, NK cells, inflammation

## Abstract

The two oncogenes *KRas* and *Myc* cooperate to drive tumorigenesis, but the mechanism underlying this remains unclear. In a mouse lung model of KRas^*G12D*^-driven adenomas, we find that co-activation of Myc drives the immediate transition to highly proliferative and invasive adenocarcinomas marked by highly inflammatory, angiogenic, and immune-suppressed stroma. We identify epithelial-derived signaling molecules CCL9 and IL-23 as the principal instructing signals for stromal reprogramming. CCL9 mediates recruitment of macrophages, angiogenesis, and PD-L1-dependent expulsion of T and B cells. IL-23 orchestrates exclusion of adaptive T and B cells and innate immune NK cells. Co-blockade of both CCL9 and IL-23 abrogates Myc-induced tumor progression. Subsequent deactivation of Myc in established adenocarcinomas triggers immediate reversal of all stromal changes and tumor regression, which are independent of CD4^+^CD8^+^ T cells but substantially dependent on returning NK cells. We show that Myc extensively programs an immune suppressive stroma that is obligatory for tumor progression.

## Highlights

• Myc and Ras cooperate *in vivo* through the tumor microenvironment
• Myc directly programs angiogenesis, inflammation, and immune suppression
• IL-23 and CCL9 are cell non-autonomous effectors of Myc-driven lung carcinogenesis
• Myc-driven tumor growth and maintenance depend on immune suppression

## Graphical Abstract

**Figure:**
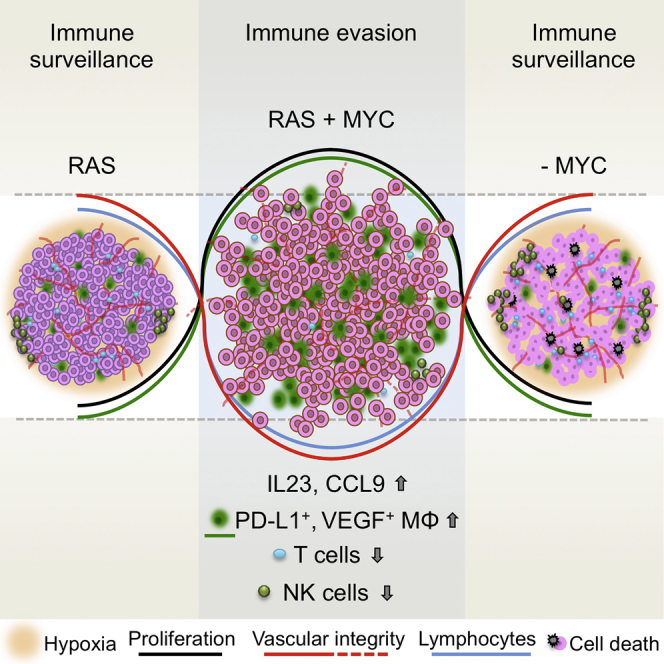

## Teaser

Oncogenic Myc activity orchestrates an immune suppressive tumor microenvironment.

## Introduction

The 1983 discovery of oncogenic cooperation between activated Ras and Myc (Land et al., 1983) demonstrated the obligate interdependence of individual oncogenic mutations. However, 30 years on, the mechanisms underlying Ras/Myc cooperation remain elusive. Early studies focused solely on Ras and Myc’s complementary cell-intrinsic outputs, such as their co-induction and/or stabilization of cell cycle proteins (Born et al., 1994, Leone et al., 1997, Wang et al., 2011) and Myc itself (Sears et al., 2000), on the reciprocal abrogation of Ras-induced senescence by Myc (Evan and Littlewood, 1998, Vaqué et al., 2005) and Ras-dependent suppression of Myc-induced apoptosis (Evan and Littlewood, 1998, Tsuneoka and Mekada, 2000), or on the capacity of Myc to overcome blockade to self-renewal in Ras-driven cells (Dong et al., 2011, Ischenko et al., 2013). However, co-transgenic *in vivo* studies in mice demonstrated that oncogenic cooperation between Ras and Myc in diverse tissues (Alexander et al., 1989, Andres et al., 1988, Boxer et al., 2004, Compere et al., 1989, Podsypanina et al., 2008, Tran et al., 2008, Yaari et al., 2005) necessarily involves modulation of interactions between the tumor cells and their stroma.

Both Ras and Myc are implicated in human non-small cell lung cancer (NSCLC). Oncogenic KRas mutations are thought to be causal drivers in ∼30% of NSCLC, especially in smokers (Rodenhuis et al., 1988), and correlate with poor prognosis (Graziano et al., 1999). Mutant KRas is principally thought to drive precocious mitogenic signaling but is also credited with reprogramming tumor cell metabolism (Kimmelman, 2015), suppressing apoptosis (Cox and Der, 2003, Kauffmann-Zeh et al., 1997) and promoting migration, metastasis (Campbell and Der, 2004), angiogenesis (Kranenburg et al., 2004, Sparmann and Bar-Sagi, 2004), and inflammation (Karin, 2005)—the latter two presumably by indirect signaling, since oncogenic Ras is confined to the epithelial tumor cell compartment. Indeed, oncogenic KRas is a potent inducer of various cytokines in many tumor types, including lung, where IL-8 (CXCL8) and IL-6 both contribute to lung cancer’s signature inflammatory phenotype (Ancrile et al., 2008, Campbell and Der, 2004, Ji et al., 2006, Kranenburg et al., 2004, Sparmann and Bar-Sagi, 2004, Sunaga et al., 2012). Aberrant Myc expression is also implicated in lung cancer. It is demonstrably overexpressed in >70% of NSCLC (Richardson and Johnson, 1993), with overt *Myc* gene amplification in the ∼20% of tumors with poorest prognosis (Iwakawa et al., 2011, Seo et al., 2014, Wolfer et al., 2010). Precocious Myc activity is causally implicated in cancers principally through its capacity to drive tumor cell proliferation; engage biosynthetic cell metabolism; and promote angiogenesis, invasion, and metastasis (Dang, 2013, Rapp et al., 2009, Shchors et al., 2006, Sodir et al., 2011, Wolfer et al., 2010). Even in NSCLC not overtly driven by mutations in Ras or Myc themselves, endogenous Ras and Myc both play prominent, even obligate, roles as downstream conduits for diverse upstream oncogenic drivers.

Here, we specifically explore the cooperative contribution made by Myc deregulation to the evolution and progression of KRas^*G12D*^-driven lung tumors *in vivo*. Using a rapidly and reversibly switchable genetic system (Murphy et al., 2008), we define the causal sequence of events by which Myc drives conversion of indolent adenomas to aggressive, inflammatory, and immune-suppressed adenocarcinomas and by which subsequent Myc de-activation triggers tumor regression. Our data show a profound role for Myc in re-programming the tumor microenvironment—especially the inflammatory and immune components of tumor stroma.

## Results

### Myc Deregulation, without Elevated Expression, Cooperates with KRas^G12D^ to Accelerate Lung Tumorigenesis *In Vivo*

To investigate Myc cooperation with KRas^*G12D*^ *in vivo*, we used mice heterozygous for the *LSL-KRas*^*G12D*^ allele (Jackson et al., 2001) and homozygous for *Rosa26-lox-STOP-lox MycER*^*T2*^ (*R26LSLMycER*^*T2*^) (Murphy et al., 2008). In these *LSL-KRas*^*G12D*^;*Rosa26-lox-STOP-lox MycER*^*T2*^ mice (hereafter referred to as *KM*), inhalation of Cre-recombinase-expressing adenovirus (AdV) triggers sporadic coincident expression in lung of oncogenic KRas^*G12D*^ from its endogenous promoter and reversibly activatable 4-OHT-dependent MycER^T2^ driven from the constitutively active *Rosa26* promoter at low, quasi-physiological levels (Murphy et al., 2008).

**Figure 1:**
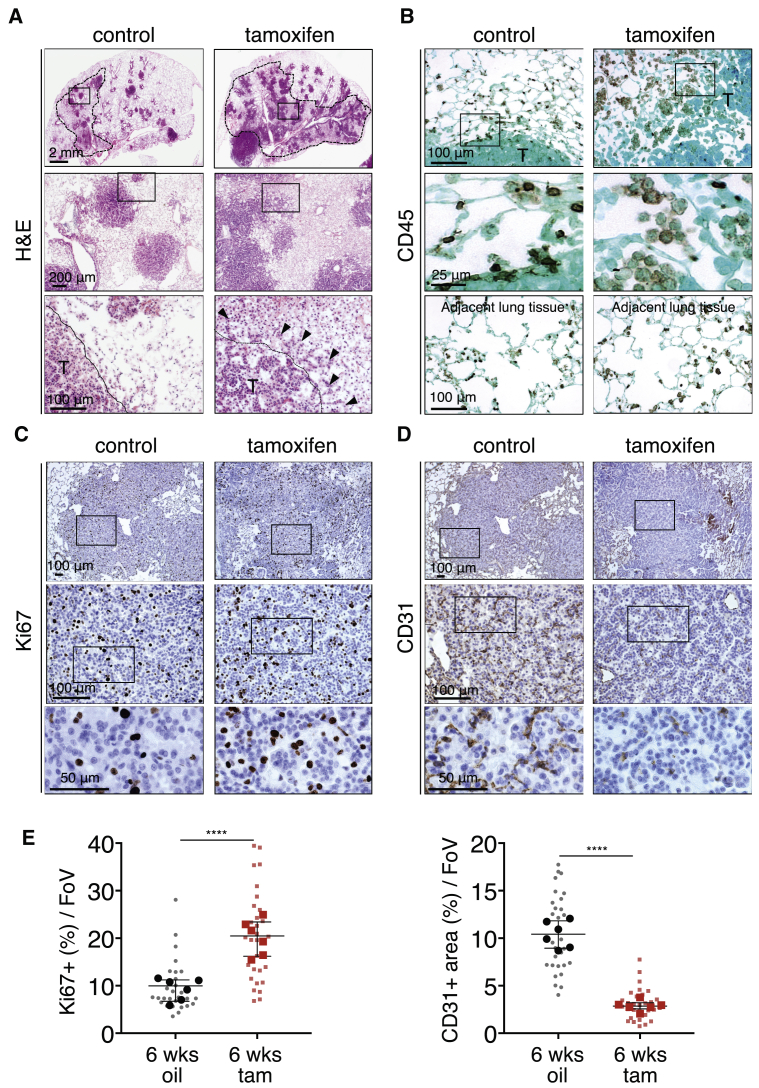
Deregulated Myc Cooperates Oncogenically with KRas^*G12D*^ in Lung (A) Representative H&E staining of lung sections 18 weeks after activation of KRas^*G12D*^ either without (control) or with (tamoxifen) Myc deregulation for the final 6 weeks. Dotted lines in top panels highlight “inflamed” regions. Boxed regions in the top row images are enlarged in the second row of panels, and boxed regions in the middle panels are further enlarged in the bottom row. T = tumor. Black arrows indicate palisades of migratory tumor cells. Scale bars are representative for rows of panels. (B–D) Representative immunostaining for the pan-leukocyte marker CD45 (B), the proliferation marker Ki67 (C) and the endothelial cell marker CD31 (D) of lung sections 12 weeks after activation of KRas^*G12D*^ either with (tamoxifen) or without (control) Myc deregulation for the final 6 weeks. Higher magnifications of the boxed areas are shown in the panels immediately below. T = tumor. Results shown in (C) and (D) are from serial sections. Scale bars are representative for rows of panels. (E) Quantification analysis of Ki67 and CD31 immunostaining of lung sections 12 weeks after activation of KRas^*G12D*^ without (6 wks oil) or with (6 wks tam) Myc activated for the last 6 weeks. FoV = field of view. n = 30 individual tumors (small symbols) from 6 total mice (large symbols) per time point. Error bars represent the median with interquartile range. p values are based on Student’s t test. ^∗∗∗∗^p < 0.0001. See also Figures S1 and S2.

**Figure S1:**
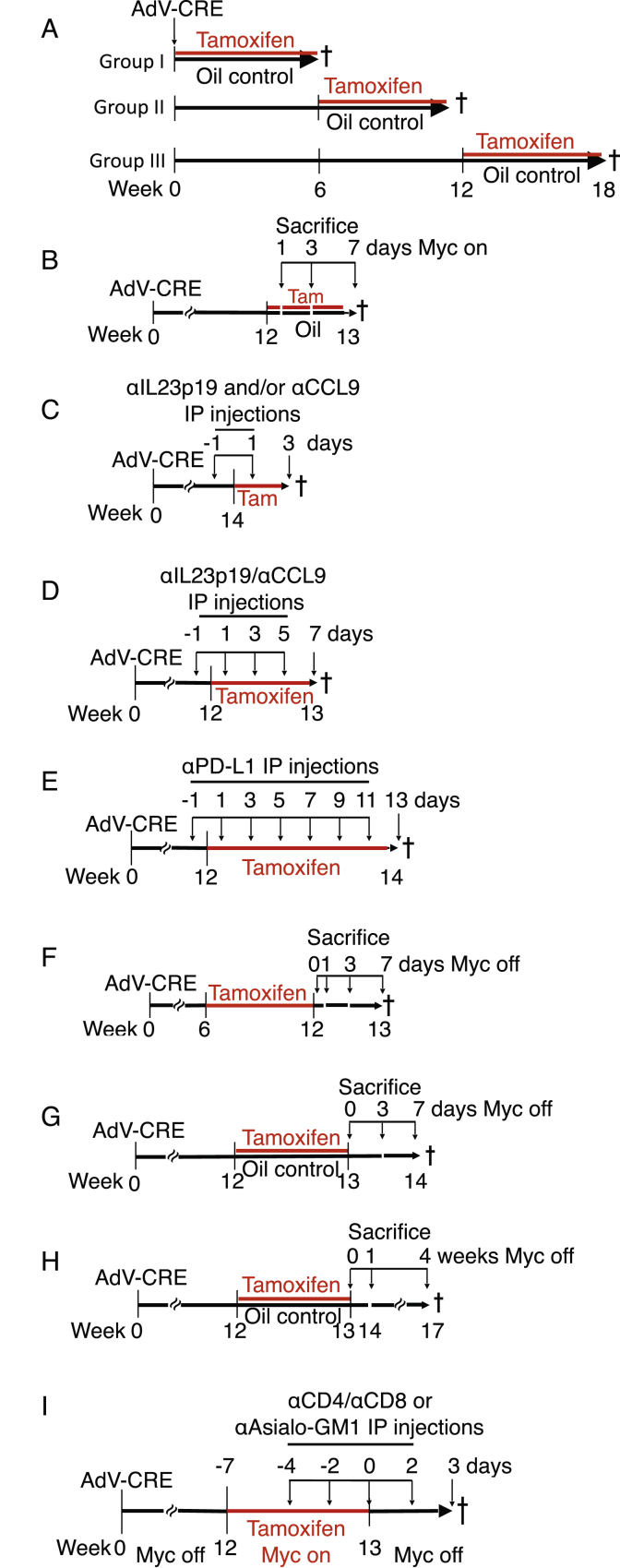
Schematic Representations of Animal Experiments, Related to Figures 1, 2, 3, 4, 5, 6, and 7 (A) Related to Figures 1 and S2. Analysis of long-term co-operation between KRas^*G12D*^ and MycER^T2^. I, II and III denote three different regimens, each with a different time points of activation of MycER^T2^ (0, 6 and 12 weeks) post-AdV-Cre activation of KRas^*G12D*^. (B) Related to Figures 2, 4, and S3. Analysis of short-term MycER^T2^ activation in KRas^*G12D*^-driven *KM* mouse lungs. 12 weeks after AdV-Cre activation of KRas^*G12D*^ in *KM* mice, MycER^T2^ was co-activated for 1, 3 or 7 days. (C) Related to Figures 3 and S4. Analysis of the impact of individual or co-blocking CCL9 and/or IL23p19 on MycER^T2^-driven *KM* lung tumor progression. 14 weeks after AdV-Cre treatment, and commencing one day prior to tamoxifen injection, mice were injected every other day for 4 days with neutralizing antibodies against either IL23p19 or CCL9 or both IL23p19 and CCL9, then euthanized. (D) Related to Figures 3 and S4. Analysis of the impact of long-term co-blocking CCL9 and/or IL23p19 on MycER^T2^-driven *KM* lung tumor progression. 12 weeks after AdV-Cre treatment, and commencing one day prior to tamoxifen injection, mice were injected every other day for 7 days with neutralizing antibodies against both IL23p19 and CCL9, then euthanized. (E) Related to Figure 4. Analysis of the impact of PD-L1 antibody blockade on MycER^T2^-driven *KM* lung tumor progression. 12 weeks after AdV-Cre administration, and commencing one day prior to tamoxifen injection to activate MycER^T2^, mice were injected every two days for two weeks with either PD-L1 neutralizing antibodies or IgG control. (F) Related to Figures 5 and S5. Analysis of short-term MycER^T2^ de-activation in *KM* lung tumors. 6 weeks after AdV-Cre activation of KRas^*G12D*^ in *KM* mice, MycER^T2^ was co-activated for 6 weeks and then de-activated for 0, 1, 3 and 7 days. (G) Related to Figure 6. Analysis of the impact of short-term activation and short-term de-activation of MycER^T2^ in KRas^*G12D*^+Myc-driven *KM* lung tumors. 12 weeks after AdV-Cre activation of KRas^*G12D*^ in *KM* mice, MycER^T2^ was co-activated for 1 week and then de-activated for 3 or 7 days. (H) Related to Figure 6. Analysis of the impact of short-term activation followed by long-term de-activation of MycER^T2^ in KRas^*G12D*^+Myc-driven *KM* lung tumors. 12 weeks after AdV-Cre activation of KRas^*G12D*^ in *KM* mice, MycER^T2^ was co-activated for 1 week and then de-activated for up to 4 weeks. (I) Related to Figures 7 and S6. Analysis of the absence of T or NK cells during short-term de-activation of MycER^T2^ in KRas^*G12D*^+Myc-driven *KM* lung tumors. 12 weeks after AdV-Cre treatment, and starting four days prior to tamoxifen injection, *KM* mice were injected every other day for four cycles with neutralizing antibodies against CD8 (or IgG control) and every four days for two cycles (at −4 and 0 day time points) with neutralizing antibodies against CD4 (or IgG control), or every other day for four cycles with neutralizing antibodies against NKp46 (anti-asialo-GM1) then sacrificed one day after the last antibody injection coincident with day 3 post MycER^T2^.

**Figure S2:**
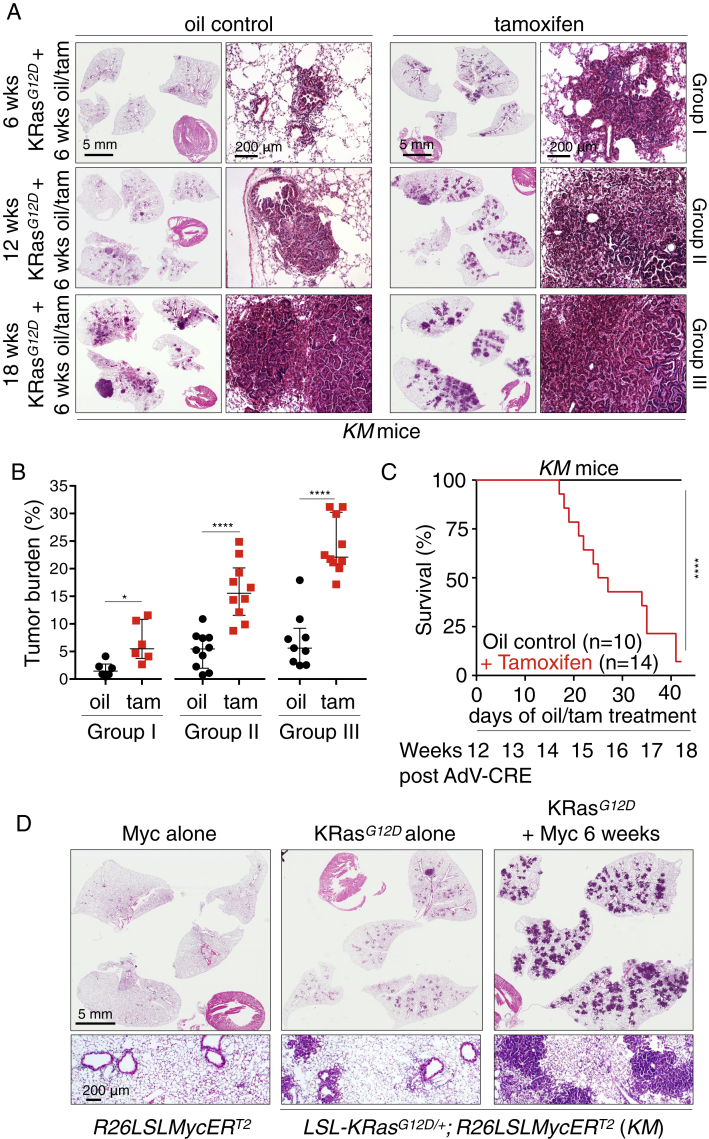
Deregulated Myc Cooperates Oncogenically with KRas^*G12D*^ at All Stages of Lung Adenoma Evolution, Related to Figure 1 (A) Representative H&E staining of lung sections harvested at 6, 12 and 18 weeks after AdV-Cre inhalation, without (oil control) or with (+ tamoxifen) Myc co-activation for the final 6 weeks, as indicated. Groups I, II and III refer to the three different experimental protocols depicted in Figure S1A. Scale bars apply to each column of panels. (B) Quantification of tumor burden in lungs of mice from Groups I, II and III, as described above. Each individual data point represents a single mouse. Group I: n = 6 mice. Group II: n = 10 mice. Group III: n = 9 (oil) and 10 (Tam) mice. (C) Kaplan-Meier survival plot of KRas^*G12D*^-driven tumor-bearing mice (12 weeks post KRas^*G12D*^ activation) then treated for 6 weeks with either oil (control) or Tamoxifen to activate Myc. n = 10 (oil) and 14 (Tam) mice. (D) Representative H&E staining of lung sections comparing the tumorigenic impact of Myc alone (left), KRas^*G12D*^ alone (middle) and Myc and KRas^*G12D*^ together (right). AdV-Cre was administered by inhalation to each of the depicted genotypes (mentioned below the panels) and lungs harvested 12 weeks later. Left - Myc alone, activated for final 6 weeks; middle - KRas^*G12D*^ alone (oil control), right – KRas^*G12D*^ plus Myc co-activated for final 6 weeks (tamoxifen treatment). Scale bars apply to each row of panels. Error bars represent the median with interquartile range. P values are based on Student’s t test (B) or Log-rank test (C). ^∗^p < 0.05, ^∗∗∗∗^p < 0.0001.

As reported (Jackson et al., 2001), activation of endogenous KRas^*G12D*^ alone in lung epithelium elicits slow outgrowth of multiple independent lesions. Multiple small foci of atypical epithelial and adenomatous hyperplasia are evident by 6 weeks after AdV-Cre inhalation, progressing to indolent and non-invasive adenomas by 12–18 weeks. Aggressive and invasive adenocarcinomas emerge sporadically much later, presumably through additional oncogenic lesions. Activation of MycER^T2^ (for 6 weeks) in 12-week-old indolent KRas^*G12D*^-driven lesions dramatically accelerated tumorigenesis over that in KRas^*G12D*^-only controls (Figures 1A, S1A, S2A, and S2B), resulting in highly proliferative, invasive tumors heavily infiltrated with leukocytes and with inchoate, nascent vasculature indicative of ongoing angiogenesis (Figures 1B–1D). Myc activation profoundly accelerated lung tumor progression at every stage of adenoma evolution (Figures S2A and S2B), triggering a precipitous drop in survival (Figure S2C). Activation of MycER^T2^ in the absence of KRas^*G12D*^ elicited no discernible lung phenotype (Figure S2D), while tamoxifen treatment alone had no effect on KRas^*G12D*^-only lung tumors (not shown). The aggressive phenotypes of KRas^*G12D*^ tumors following MycER^T2^ activation were indistinguishable from those of KRas^*G12D*^ tumors driven by constitutive *Rosa26*-driven Myc (not shown).

### Myc Deregulation in Tumor Cells Rapidly Induces Diverse Stromal Changes

**Figure 2:**
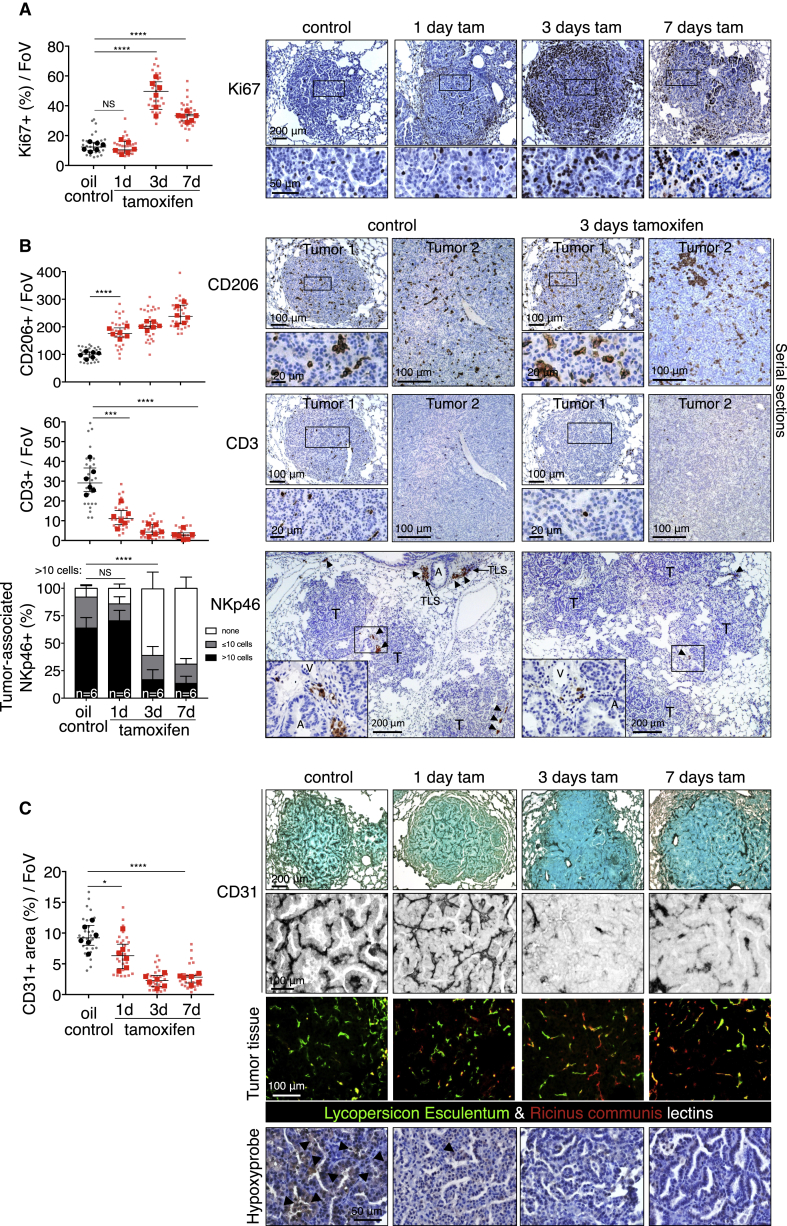
Deregulation of Myc in Epithelial Adenoma Cells Immediately Re-programs the Tumor Stroma (A) Quantitative and representative immunohistochemical analysis of Ki67 expression at indicated time points (0, 1, 3, and 7 days) after activation of Myc (tam) compared to KRas^*G12D*^-only (oil-treated control). Boxed regions are magnified below. Scale bars apply across each row. (B) Quantification and representative immunostaining for the activated macrophage marker CD206, the T cell marker CD3, and the NK cell marker NKp46 on sections of KRas^*G12D*^-driven adenomas at indicated time points after Myc activation (tamoxifen). Two representative individual tumors from serial sections are shown for CD206 and CD3, and quantitation is restricted to the areas within tumor boundaries. Boxed regions are shown at higher magnification directly below. NK cells cluster principally in juxta-tumoral tertiary lymphoid structures (TLSs), and/or adjacent to large tumor-associated blood vessels (V) and airways (A). Hence, quantitation of NK cells includes both tumor area and tumor-associated vasculature and TLSs. For NK cell staining (NKp46), lung regions with multiple tumors are shown and higher magnifications of boxed regions are displayed as insets. Arrows indicate NK cells occupying tertiary lymphoid structure (or part thereof) or residing next to tumor-associated blood vessel and/or airway. T = tumor. (C) Quantification and representative immunostaining using the endothelial cell marker CD31 (top two rows) in lung tumor tissue isolated from mice described in (A). Grayscale insets are magnified from the panels immediately above. (Third row) Visualization of vascular integrity and permeability in tumors—using fluorescein isothiocyanate (FITC)-conjugated *Lycopersicon esculentum* lectin (green), which binds to the luminal surface of all blood vessels, and rhodamine-conjugated *Ricinus communis* agglutinin I (red), which binds to the endothelial basement membrane only of leaky nascent vessels—at indicated time points after Myc activation (tamoxifen). (Bottom row) Tumor hypoxia assessed by hypoxyprobe immunostaining. Black arrows indicate hypoxic regions. Scale bars apply across each row. Quantification graphs: FoV = field of view. n = 30 individual tumors (small symbols) from 6 mice (large symbols) per time point. Error bars represent the median with interquartile range (Ki67, CD206, CD3, CD31) or mean ± SD (NKp46). p values are based on Student’s t test (Ki67, CD206, CD3, CD31) or two-way ANOVA (NKp46). NS = non-significant; ^∗^p < 0.05, ^∗∗∗^p < 0.001, ^∗∗∗∗^p < 0.0001. See also Figures S1, S3, and S7.

**Figure S3:**
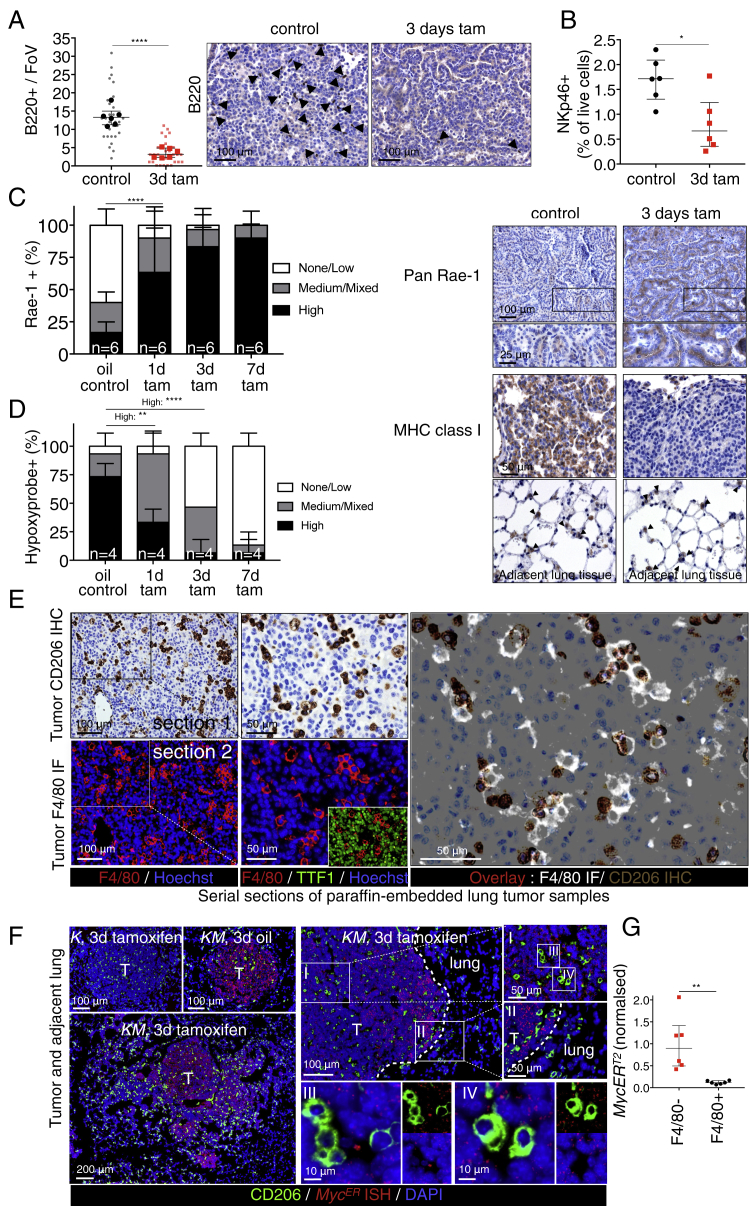
Deregulated Myc Rapidly Re-programs Tumor Immunity, Related to Figure 2 (A) Quantification and representative immunostained images for B lymphocytes (anti-B220 *aka* CD45R) in mice treated with tamoxifen for 3 days. (B) Quantification (by flow cytometry) of NKp46 positive cells as percentage of total live cells isolated from tumor-laden whole lungs of mice treated with oil (KRas^*G12D*^-only) or 3 days tamoxifen (+ Myc). n = 6 mice per time point. (C) Quantitative analysis (left) and representative immunostaining (right) of NK cell-activating signals showing rapid upregulation of the NKG2D-ligand Rae-1 (PAN Rae-1 antibody) and downregulation of MHC Class I on lung tumor cells following Myc activation. Bottom panels show MHC Cass I expression on adjacent normal lung epithelium (closed arrows). Scale bars apply across each row. (D) Quantification of hypoxyprobe staining shown in Figure 2C (bottom row), confirming rapid transition from hypoxia to normoxia in adenomas following Myc activation. (E) Immunohistochemistry and immunofluorescence analysis of serial sections of paraffin-embedded lung tumors of *KM* mice showing concordance of CD206 and F4/80 immunostaining. *KM* mice were treated for three days with Tamoxifen, lung tissue harvested and serial sections taken for immunostaining. Top row: representative Immunostaining for macrophage marker CD206 with boxed region enlarged immediately to the right. Bottom row: Immunofluorescence staining for macrophage marker F4/80 with boxed region enlarged immediately to the right. Inset in bottom right panel shows non-overlap of staining for F4/80 and for the epithelial cell marker TTF1. Right large panel: overlay of Immunofluorescence F4/80 and immunohistochemistry of CD206 showing overlap. (F) Epithelial fluorescence *in situ* hybridization of *MycER*^*T2*^ (red) combined with immunofluorescence for lung macrophage CD206 (green). Representative pictures are shown. Left panels: KRas^*G12D*^-only mice (*K*) mice express no detectable *MycER*^*T2*^ whereas *KM* mice show clear *MycER*^*T2*^ nuclear staining confined to tumor masses that is independent of *MycER*^*T2*^ activation (tamoxifen treatment). Right Panels: I and II and their progressive enlargements show *MycER*^*T2*^ expression is specific to tumor cells and absent from normal lung epithelial cells. Dotted line indicates tumor boundary. III and IV confirm absence of any detectable *MycER*^*T2*^ in CD206^+^ macrophages. (G) *MycER*^*T2*^ RNA expression in F4/80-affinity isolated lung macrophages in *KM* mice 16 weeks post AdV-CRE. Results are normalized to the average of the F4/80^-^ fraction. n = 6 mice per fraction (F4/80^-^ versus F4/80^+^). Quantification graphs: FoV = Field of View. (A): n = 30 individual tumors (small symbols) from 6 mice (large symbols) per time point. (C): n = 4 mice per time point, n = 20 tumors analyzed; 5 per mouse. (D): n = 6 mice per time point, n = 30 tumors analyzed; 5 per mouse. Error bars represent the median with interquartile range (B220, NKp46) or mean ± SD (Rae-1, Hypoxyprobe). P values are based on Student’s t test (B220, NKp46) or two-way ANOVA (Rae-1, Hypoxyprobe). ^∗^p < 0.05, ^∗∗^p < 0.01, ^∗∗∗∗^p < 0.0001.

The lung tumors resulting from long-term combined KRas^*G12D*^ and Myc activity are more advanced and aggressive than the indolent tumors driven by KRas^*G12D*^ alone, being markedly more proliferative, invasive, inflammatory, and angiogenic. Such dramatic stromal changes could either be indirect, passive consequences of the disruption of normal lung architecture caused by Myc-driven tumor growth or, alternatively, direct proximal consequences of Myc activation within the adenoma epithelial compartment. Hence, to determine how Myc activation causally elicits its diverse phenotypic changes, we used the rapid, synchronous, and reversible *in vivo* activation possible with MycER^T2^ (Pelengaris et al., 2002, Shchors et al., 2006, Sodir et al., 2011). Matched cohorts of adult *KM* mice were infected with AdV-Cre, and 12 weeks later, tamoxifen was systemically administered to activate Myc in the epithelial cells of the incipient lung adenomas (Figure S1B). Myc activation triggered proliferation broadly across the whole adenoma mass (Figure 2A), as well as immediate and widespread changes in the tumor-associated stroma. Within 24 hr of Myc activation, we saw a dramatic influx of CD206^+^ macrophages (Figure 2B) into tumor masses and concurrent loss of CD3^+^ T (Figure 2B) and B220^+^ B cells (Figure S3A). We also saw a rapid decline of NKp46^+^ natural killer (NK) cells (Figure 2B) from the juxta-tumoral blood vessels and tertiary lymphoid structures (TLS) to which they principally localize (Fridman et al., 2012, Goc et al., 2013, Joshi et al., 2015) and a fall in total NK cell numbers in the entire lungs (Figure S3B). This rapid expulsion of NK cells is especially intriguing given that Myc activation also triggered a profound upregulation of Rae-1 NKG2D ligands and downregulation of major histocompatibility complex (MHC) class I (Figure S3C)—both potent activating signals for NK-like cells (Morvan and Lanier, 2016)—in lung adenoma cells. Finally, Myc activation triggered the abrupt onset of angiogenesis, marked by loss of vessel integrity, increased vessel leakiness, and relief of the widespread hypoxia characteristic of the original indolent adenomas (Figures 2C and S3D). All of these diverse changes in tumor and stroma preceded overt Myc-induced tumor expansion, and all were sustained so long as Myc activity was maintained (see below).

### Myc-Induced Stromal Changes in Lung Tumors Are Instructed by CCL9 and IL-23

To exclude the possibility that some of the phenotype we observe might be a consequence of Cre induction of MycER^T2^ in long-lived resident alveolar macrophages we directly assessed whether MycER^T2^ is expressed in the lung tumor-associated macrophages using two independent macrophage markers co-expressed on lung tumor-associated macrophages (Figure S3E)—the mannose receptor CD206 and EMR1, a GPCR family adhesion molecule recognized by the F4/80 antibody. First, we showed that abundant nuclear *in situ MycER*^*T2*^ RNA signals, while evident across the entire adenoma epithelial compartment of KM mice, are entirely absent from all CD206^+^ macrophages (Figure S3F). Second, analysis of lung macrophages harvested from disaggregated *KM* mouse lungs and purified by F4/80 antibody affinity showed complete absence of *MycER*^*T2*^ mRNA expression, all of which was confined to the F4/80 negative lung cell fraction (Figure S3G). Hence, none of the stromal changes we observe are the result of direct activation of MycER^T2^ in macrophages.

**Figure 3:**
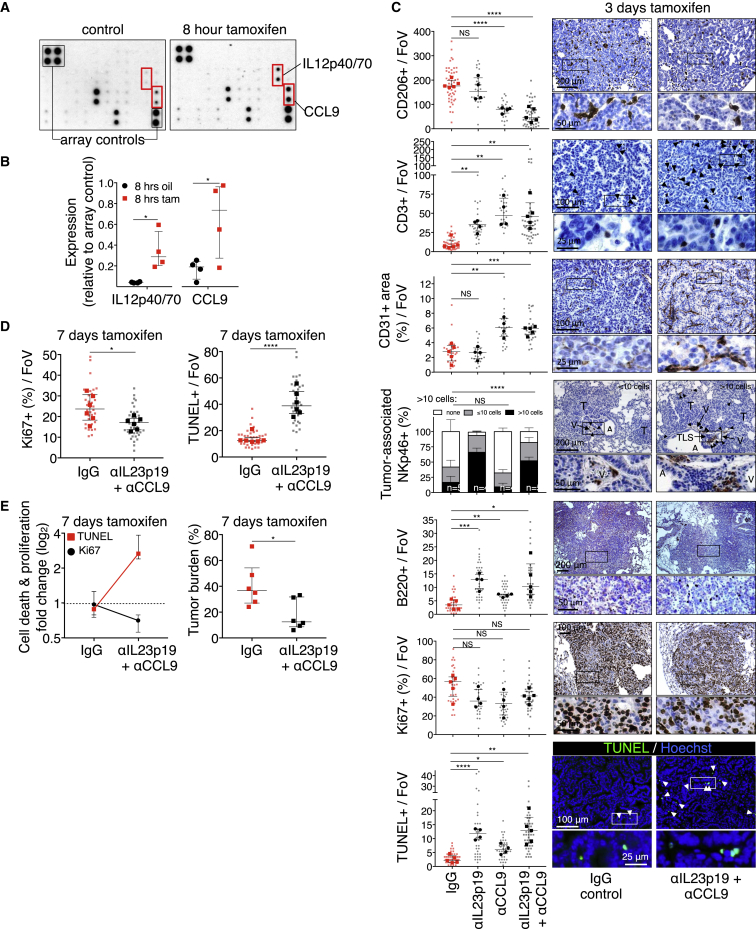
IL-23 and CCL9 Mediate Myc-Induced Remodeling of Lung Tumor Stroma (A) Representative inflammation antibody arrays probed with whole-lung protein lysates from mice treated for 8 hr with oil (control) or tamoxifen to activate Myc. (B) Quantification of IL12p40/70 and CCL9 signals derived from arrays shown in (A). Each individual data point represents a single mouse. (C) Quantification and representative examples of immunostaining for CD206, CD3, CD31, NKp46, B220, Ki67, and TUNEL after Myc activation for 3 days in mice sham treated (IgG control), treated with either IL23p19- or CCL9-blocking antibodies, or co-treated with both IL23p19- and CCL9-blocking antibodies. Boxed areas in each image are shown enlarged in the panels directly below. Scale bars apply across each row. (D) Quantification of tumor cell proliferation (Ki67, left) and cell death (TUNEL, right) after Myc activation for 7 days (with tamoxifen) in mice co-treated with either IgG control or co-treated with IL23p19- and CCL9-blocking antibodies. (E) Quantification of fold change in tumor cell death (TUNEL) and proliferation (Ki67) (left) and tumor burden (right) after Myc activation for 7 days (with tamoxifen) in mice co-treated with either IgG control or co-treated with IL23p19- and CCL9-blocking antibodies. Quantification graphs: FoV = field of view. Small symbols = individual tumors, large symbols = average per mouse. (C) n = 4 mice and n = 25–35 tumors (individual anti-IL23p19 or anti-CCL9 treatment) or n = 5 mice and n = 30–50 tumors (IgG control or anti-IL23p19 and anti-CCL9 co-treated). (D) n = 30 tumors from 6 mice treatment point. (E) n = 6 mice per treatment group. Error bars represent the median with interquartile range. p values are based on Student’s t test (CD206, CD3, CD31, B220, Ki67, TUNEL) or two-way ANOVA (NKp46). NS = non-significant; ^∗^p < 0.05, ^∗∗^p < 0.01, ^∗∗∗^p < 0.001, ^∗∗∗∗^p < 0.0001. See also Figures S1, S4, and S7.

**Figure S4:**
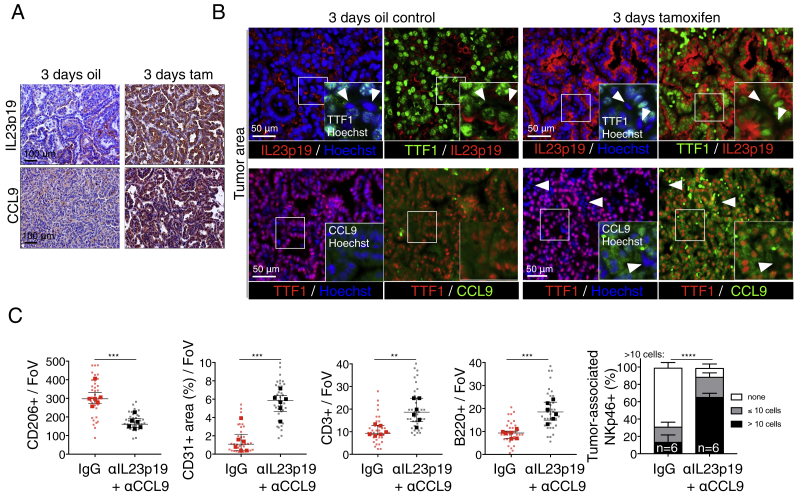
Myc Induces IL-23 and CCL9 in the Epithelial Compartment of Lung Adenomas and Their Continued Expression Is Required to Maintain an Immune-Suppressed Phenotype, Related to Figure 3 (A) Representative immunostaining showing induction of IL23p19 and CCL9 following activation of Myc (3d tam), compared to KRas^*G12D*^-only control (3d oil). Scale bars apply to panels in each column. (B) Immunofluorescence analysis showing coincident staining of Myc-induced cell-surface IL23p19 and cytoplasmic CCL9 with the bronchoalveolar nuclear marker TTF1 in tumors following activation of MycER^T2^ (3d tamoxifen), compared to KRas^*G12D*^-only control (3d oil control). Insets show magnified images of each boxed region. White arrows indicate cells negative for both TTF1 and IL23p19 (top row) or both TTF1 and CCL9 (bottom row). (C) Quantification of immunostaining for lung tumor macrophages (CD206), vascular endothelial cells (CD31^+^), T cells (CD3^+^), B cells (B220^+^) and NK (NKp46^+^) cells after Myc activation for 7 days in mice coincidentally treated either with control (IgG) or co-treated with IL23p19- and CCL9-blocking antibodies. FoV = Field of View. n = 30 individual tumors (small symbols) from 6 mice (large symbols) per treatment group. Error bars represent the median with interquartile range. P values are based on Student’s t test (CD206, CD31, CD3, B220) or two-way ANOVA (NKp46). ^∗∗^p < 0.01, ^∗∗∗^p < 0.001, ^∗∗∗∗^p < 0.0001.

As MycER^T2^ is activated only in the epithelial compartment of the *KM* lung adenomas, the rapid stromal changes that Myc elicits must be mediated by signals released from those epithelial cells. To identify them, we activated MycER^T2^ in adenoma-bearing *KM* mice, harvested their lungs after 8 hr, and used whole-lung protein extracts to interrogate an inflammation antibody array. Of 40 potential candidates, only two inflammatory signals were detectably upregulated by Myc: the chemokine CCL9 (a.k.a. MIP-1γ) and the p40 subunit common to IL-12 and IL-23 (Figures 3A and 3B). This upregulation was confirmed immunohistochemically: by 3 days post Myc activation, intense cytoplasmic CCL9 immunostaining and cell surface immunostaining for the p19 subunit of IL-23 were evident (Figure S4A) in TTF1^+^ epithelial cells (Stenhouse et al., 2004) while absent from both non-tamoxifen-treated controls (Figure S4B) and tamoxifen-treated KRas^*G12D*^-only-driven adenomas (not shown). The extremely rapid induction of both CCL9 and IL-23 following epithelial Myc activation, plus their histological co-localization, confirm the adenoma epithelial compartment as the source of CCL9 and IL-23 production and secretion.

To determine whether CCL9 and IL-23 release is the proximal trigger for the stromal changes that Myc activation elicits, adenoma-bearing *KM* mice were pre-treated with CCL9- and/or IL-23-blocking antibodies and Myc then activated by tamoxifen administration (Figures S1C and S1D). Co-blockade of both CCL9 and IL-23 sharply reduced Myc-induced macrophage influx; blocked the Myc-induced angiogenic switch; and forestalled the loss of T, B, and NK cells (Figures 3C and S4C). And while Myc-induced tumor cell proliferation was only modestly suppressed, it was now accompanied by significant tumor cell apoptosis (Figure 3C), resulting in significant reduction in tumor burden by 7 days’ blockade (Figures 3D and 3E). To define the individual roles of CCL9 and IL-23 in mediating Myc-induced stromal changes, we used antibodies to inhibit each signal individually. Blockade of IL-23 had no discernible impact on either Myc-induced macrophage influx or angiogenesis but profoundly blocked Myc-induced loss of T, B, and NK cells; weakly suppressed tumor cell proliferation; and potently exacerbated tumor cell apoptosis (Figure 3C). By contrast, blockade of CCL9 alone profoundly inhibited Myc-induced macrophage influx, angiogenesis, and T cell loss but had no impact on Myc-driven NK cell exclusion, weakly inhibited B cell loss, and only modestly inhibited tumor cell proliferation or exacerbated apoptosis (Figure 3C).

**Figure 4:**
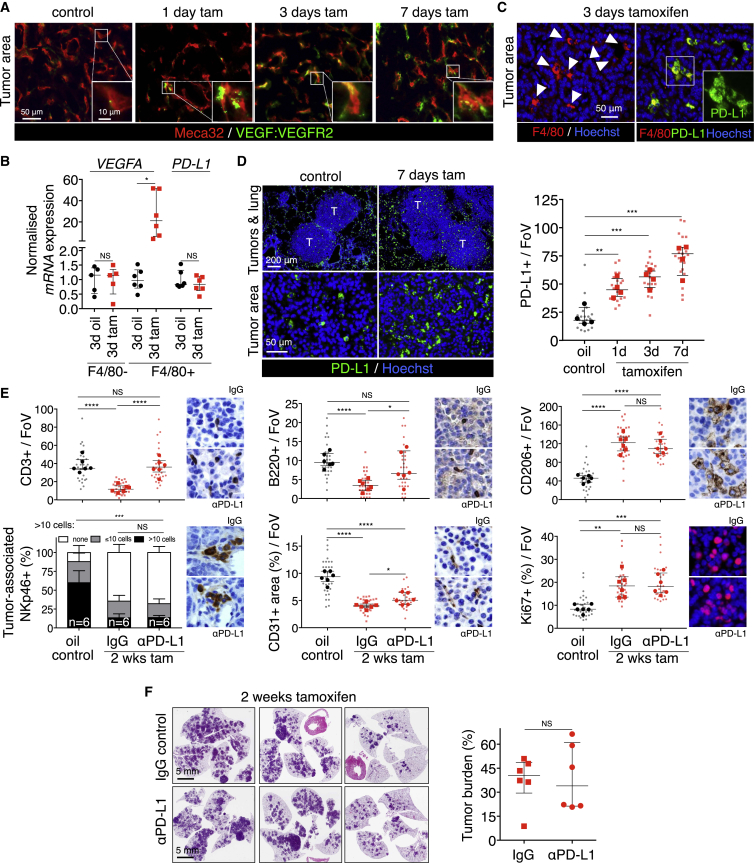
Macrophages Mediate Myc-Induced Angiogenesis and T Cell Exclusion (A) Coincident immunostaining of Meca32 and receptor-bound VEGF (VEGF:VEGFR2) in tumors at indicated time points (1, 3, and 7 days) after activation of Myc by tamoxifen treatment compared to KRas^*G12D*^-only (control). Inset shows enlargement of regions boxed in white. Scale bars apply across each row. (B) Quantitative RT-PCR for *VEGFA* and *PD-L1* mRNA in F4/80^+^ macrophages and F4/80^−^ lung cells derived from whole tumor-laden lungs following 3 days Myc activation (tam) versus to KRas^*G12D*^-only control (3d oil). (C) Immunofluorescence analysis of F4/80 and PD-L1 in lung tumors following Myc activation. Scale bar applies to both large panels. White arrows indicate F4/80^+^ cells. (D) Immunostaining and respective quantification of PD-L1-positive macrophages in tumors after Myc activation for indicated time points (tamoxifen). T = tumor. Scale bars apply across each row. (E) Quantification of immunohistochemical analysis for CD3, B220, CD206, NKp46, CD31, and Ki67 of lung tumors after Myc activation for 2 weeks concurrently with systemic treatment of PD-L1 blocking antibody compared to KRas^*G12D*^-only tumors (oil control). (F) H&E staining (left) and quantification (right) of lung tumor burden in mice treated concurrently with tamoxifen (to activate Myc) and either control IgG or PD-L1-blocking antibody. Each individual data point represents a single mouse (n = 6 mice per group). Quantification graphs: FoV = field of view. Small symbols = individual tumors, large symbols = average per mouse. (B) Each individual data point represents a single mouse. n = 5 (F4/80^−^; *VEGFA*), or 6 (F4/80^+^; *VEGFA*, *PD-L1*), and shows the expression data normalized to the average of the respective oil control. (D) n = 20 individual tumors from 4 mice per treatment group. Error bars represent the median with interquartile range. (E) n = 30 individual tumors from 6 mice per treatment group. Error bars represent the median with interquartile range (CD3, CD206, B220, CD31, Ki67) or mean ± SD (NKp46). p values are based on Student’s t test (CD3, B220, CD206, CD31, Ki67) or two-way ANOVA (NKp46). NS = non-significant; ^∗^p < 0.05, ^∗∗^p < 0.01, ^∗∗∗^p < 0.001, ^∗∗∗∗^p < 0.0001. See also Figures S1 and S7.

To dissect further the causal sequence by which CCL9 and IL-23 modify lung tumor stroma, we next examined the mechanism behind Myc-induced angiogenesis. The rapid remodeling of tumor vasculature that Myc triggers is a prototypical effect of VEGF, and indeed, immunohistochemistry using the GVM39 monoclonal antibody that specifically recognizes VEGF bound to its cognate VEGFR2 receptor (Brekken et al., 1998) indicated rapid VEGF engagement of endothelial cell VEGFR2 following Myc activation (Figure 4A). To identify the source of VEGF, we assayed *VEGFA* expression in F4/80-affinity-isolated lung macrophages, since macrophages are established drivers of angiogenesis in diverse tumors (Pollard, 2004). *VEGFA* mRNA expression was evident in the F4/80^+^ cell fraction of tamoxifen-treated *KM* lungs (Figure 4B), but only after MycER^T2^ activation. By contrast, expression of PD-L1, constitutive in F4/80^+^ *KM* lung macrophages (Igarashi et al., 2016), was unaffected by Myc activation status (Figure 4B). Together with our observation that Myc-driven *KM* lung tumor angiogenesis is completely absent when macrophage influx is abrogated by CCL9 blockade, our data identify macrophage-derived VEGF as the principal instigator of Myc-induced lung adenoma angiogenesis.

Since CCL9 blockade prevented Myc-induced loss of T cells from *KM* adenomas, we hypothesized that incoming macrophages might mediate T cell exclusion. Immunostaining showed PD-L1 expression on lung macrophages but absent from the adenoma epithelium, irrespective of Myc status (Figure 4C). After Myc activation, F4/80^+^ PD-L1^+^ macrophages rapidly accumulated in the tumors (Figure 4D), coinciding temporally with loss of CD3^+^ T cells (see Figure 2B). To test directly whether accumulation of PD-L1-positive macrophages was causally responsible for the T cell loss, we activated MycER^T2^ in adenoma-bearing *KM* mice while concurrently administering PD-L1-blocking immunoglobulin (Figure S1E). PD-L1 blockade completely abolished Myc-induced T cell loss and partially suppressed B cell loss but had no discernible impact on Myc-induced macrophage influx, angiogenesis, or the rapid decline in juxta-tumoral NK cells (Figure 4E). Moreover, PD-L1 blockade—and consequent persistence of T cells—had no measurable inhibitory impact on tumor cell proliferation (Figure 4E), tumor growth, or tumor burden (Figure 4F).

Taken together, our data delineate a causal sequence whereby Myc activation in lung epithelial adenoma cells triggers rapid release of IL-23 and CCL9. IL-23 drives the rapid exit of T, B, and NK cells, and since IL-23 blockade triggers tumor cell apoptosis, we infer its activity is required for outgrowth of Myc-driven lung adenocarcinomas. CCL9 is principally responsible for the rapid recruitment of PD-L1^+^ macrophages into the tumor environment whose rapid induction and release of VEGF drives the CCL9-dependent angiogenic switch while the PD-L1 they express is, along with IL-23, required for the rapid loss of T, but not of NK, cells.

### Lung Tumors, and Their Associated Stroma, Rapidly Acquire Dependency upon Myc Activity for Their Maintenance

Previous transgenic studies of tumors co-driven by oncogenic Ras and Myc concur that such tumors are dependent on both oncogenes for their maintenance. Acquisition of such dependence, sometimes dubbed “oncogene addiction,” is a widely reported phenomenon, although its underlying mechanistic basis remains unknown.

**Figure 5:**
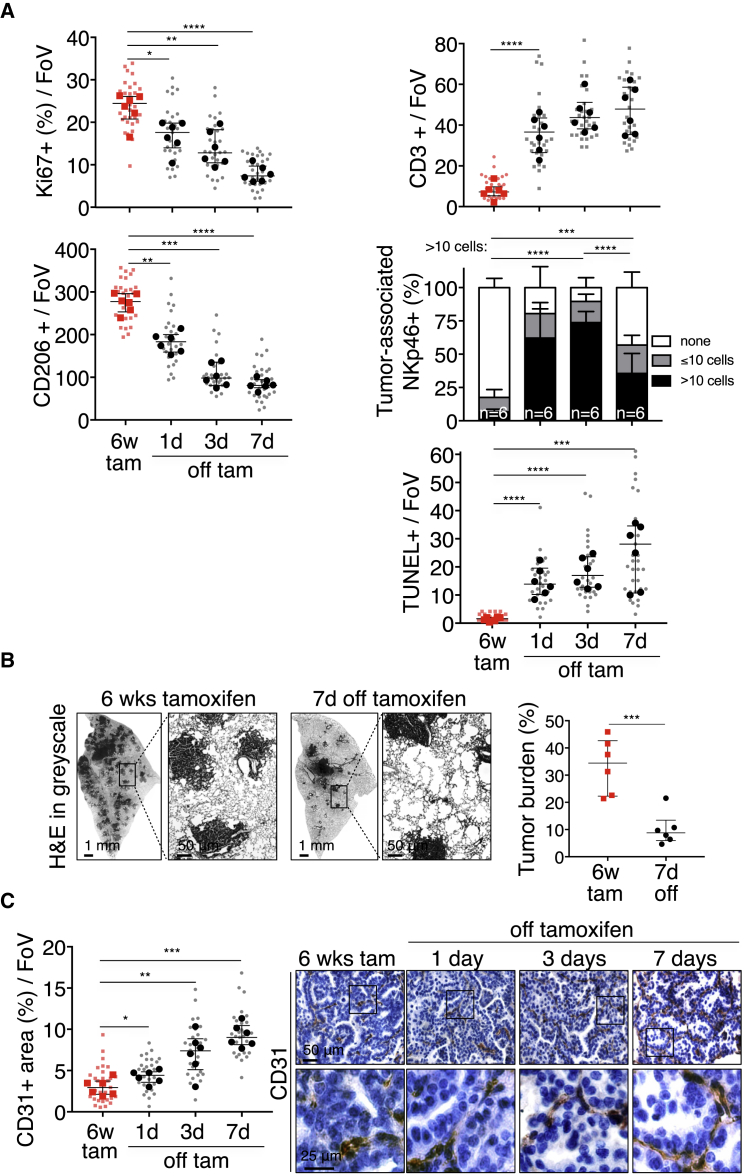
Myc De-activation Triggers Immediate Collapse of Myc-Induced Stroma Together with Tumor Regression (A) Quantification of immunostaining for Ki67, CD206, CD3, NKp46, and TUNEL in adenocarcinomas at indicated times after Myc de-activation (off tam) compared to Myc activation for 6 weeks (6w tam). (B) (Left) Representative lung lobes of tumor load 7 days after Myc de-activation (6w tamoxifen treatment versus 7d off tamoxifen). Higher magnification of boxed regions in panel to their right. (Right) Quantification of lung tumor burden in mice treated with tamoxifen for 6 weeks (6w tam) followed by withdrawal of treatment for 7 days (7d off). (C) Quantification and representative CD31 immunostaining at indicated times following Myc de-activation (off tam) compared to Myc activation for 6 weeks (6w tam). Boxed areas in each image are shown enlarged in the panels directly below. Scale bars apply across each row. Quantification graphs: FoV = field of view. (A and C) n = 30 individual tumors (small symbols) from 6 mice (large symbols) per time point. (B) 6 mice per time point. Error bars represent the median with interquartile range (Ki67, CD206, CD3, TUNEL) or mean ± SD (NKp46). p values are based on Student’s t test (Ki67, CD206, CD3, TUNEL) or two-way ANOVA (NKp46). ^∗^p < 0.05, ^∗∗^p < 0.01, ^∗∗∗^p < 0.001, ^∗∗∗∗^p < 0.0001. See also Figures S1 and S5

**Figure S5:**
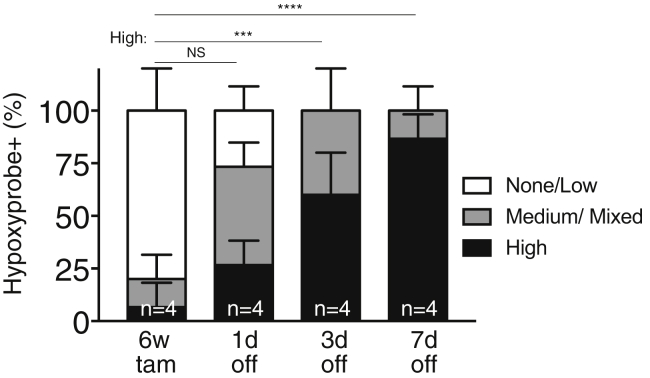
Myc De-activation Immediately Reverses Normoxia in *KM* Lung Tumors, Related to Figure 5 Quantification of immunostaining for Hypoxyprobe in adenocarcinomas at indicated times after Myc de-activation (6w tam versus 1, 3, 7 days off). FoV = Field of View. n = 4 mice and n = 20 tumors per time point; 5 tumors were analyzed per mouse. Error bars represent mean ± SD. P values are based on two-way ANOVA. NS = non-significant, ^∗∗∗^p < 0.001, ^∗∗∗∗^p < 0.0001.

To address whether KRas^*G12D*^-driven lung tumors acquire dependency on Myc, we activated MycER^T2^ in adenoma-bearing *KM* mice for 6 weeks and then de-activated it by tamoxifen withdrawal (Figure S1F) (Wilson et al., 2014). Myc de-activation triggered an immediate drop in tumor cell proliferation, rapid exit of macrophages and re-entry of CD3^+^ T cells, an influx of NKp46^+^ NK cells, and induction of tumor apoptosis (Figure 5A) and tumor regression, evident by the appearance of voids within the tumor masses and profound drop in overall tumor burden (Figure 5B). Concurrently, tumor blood vessels “normalized” (Figure 5C) and hypoxia rose (Figure S5).

**Figure 6:**
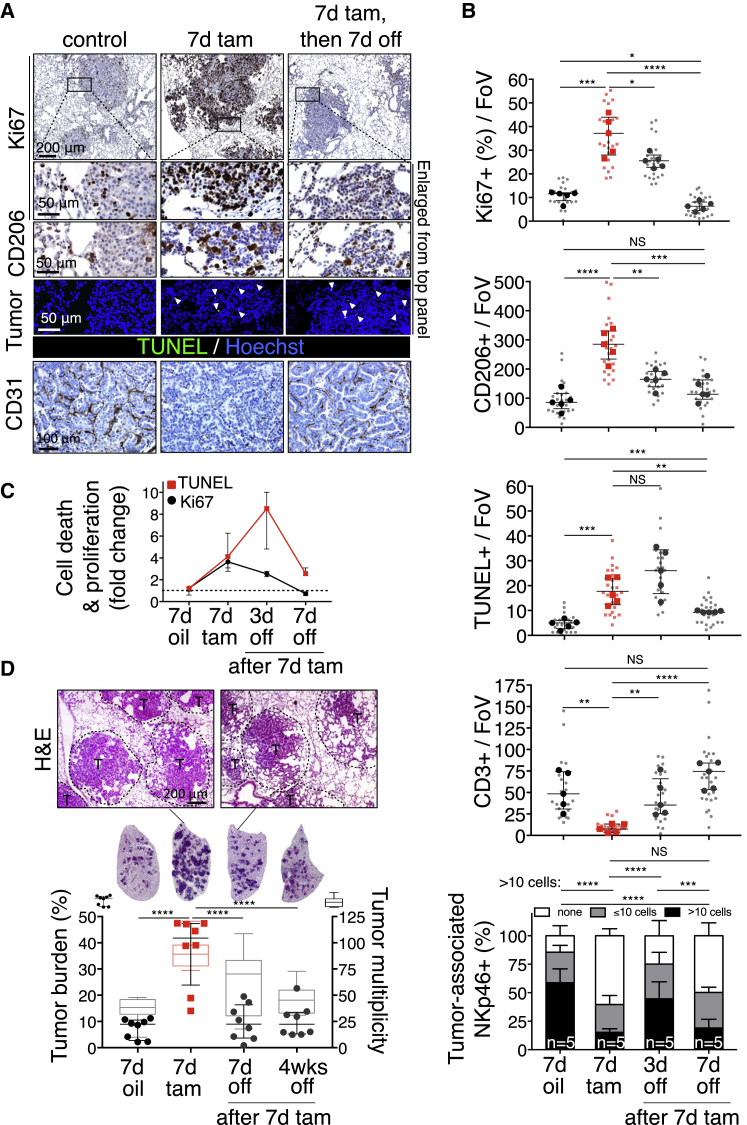
Dependency on Myc is Rapidly Acquired by Lung Adenocarcinomas (A) Representative immunostaining for Ki67, CD206, TUNEL (white arrows), and CD31 in adenocarcinomas at indicated time points following Myc activation for 7 days (7d tam) and subsequent de-activation (7d tam, then 7d off) compared to KRas^*G12D*^-only (control). The tumor edges boxed in the top panels are shown enlarged below. Scale bars apply across each row. (B) Quantification of histological changes in Ki67, CD206, TUNEL, CD3, and NKp46 in lung tumors after short term (7d tam) Myc-activation and subsequent Myc de-activation for 3 and 7 days (3d, 7d off) compared to KRas^*G12D*^-only control (7d oil). (C) Fold change in tumor cell proliferation (Ki67) and death (TUNEL) in mice treated with oil (control) or with tamoxifen for 7 days followed by tamoxifen withdrawal for 3 or 7 days. Individual and average values of tumor cell death and proliferation are displayed in (B). (D) Representative H&E staining of part of and whole lung lobes together with corresponding quantification of total tumor burden (left y axis) and tumor multiplicity (right y axis) in lungs of mice treated with tamoxifen for 7 days followed by Myc de-activation for either 7 days or 4 weeks (7d, 4w off) and compared to KRas^*G12D*^-only control (7d oil). T = tumor. Quantification graphs: FoV = field of view. (B): n = 25 individual tumors (small symbols) from 5 mice (large symbols) per time point. (D) Each individual data point represents a single mouse (n = 8 mice per group). Box and whisker graphs represent tumor multiplicity of 8 mice per group. Error bars represent the median with interquartile range (Ki67, CD206, TUNEL, CD3) or mean ± SD (NKp46). p values are based on Student’s t test (Ki67, CD206, TUNEL, CD3) or two-way ANOVA (NKp46). NS = non-significant; ^∗^p < 0.05, ^∗∗^p < 0.01, ^∗∗∗^p < 0.001, ^∗∗∗∗^p < 0.0001. See also Figure S1.

To ascertain how rapidly Myc dependency arises, Myc was activated in adenoma-bearing *KM* mice for 7 days—sufficient time to induce all stromal changes and for significant increase in tumor load (Figures 6A and S1G). De-activating Myc, even after this short period of Myc activity, triggered an immediate drop in tumor cell proliferation, rapid efflux of macrophages, normalization of vasculature, and repopulation by T and NK cells (Figures 6B and 6C). Hence, the dependency of KRas^*G12D*^-driven lung adenocarcinomas on deregulated Myc activity arises immediately across the whole tumor mass and across all tumors. Of note, however, Myc de-activation failed to drive complete tumor regression; rather, the tumors regressed back to their previously indolent KRas^*G12D*^-only adenoma state and thereafter persisted indefinitely (Figures 6D and S1H).

**Figure 7:**
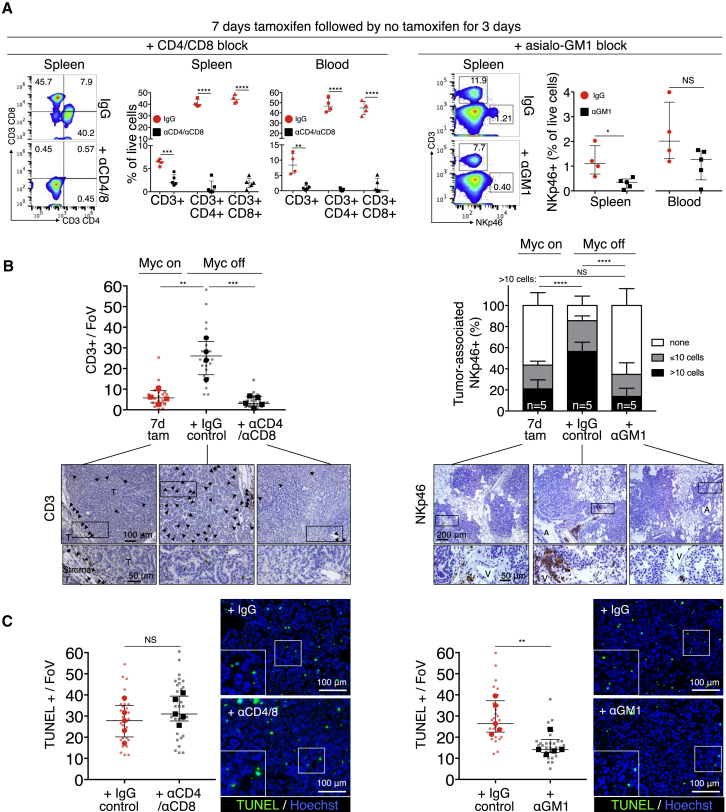
Depletion of NKp46^+^ Cells, but Not of CD4^+^ and CD8^+^ T Cells, Retards Tumor Regression following Myc De-activation (A) Flow cytometric quantification of CD3^+^, CD4^+^, and CD8^+^ T cells and NKp46^+^ NK cells in spleen and blood after systemic administration of, respectively, CD4/CD8 (left)- or asialo-GM1 (right)-blocking antibodies in mice up to 3 days after 7 days of tamoxifen (Myc on) treatment. Representative flow profiles of spleen CD3^+^/CD4^+^, CD3^+^/CD8^+^, NKp46^+^, and CD3^+^ cells are shown. (B) (Left) Quantitation of immunostaining for CD3^+^ T cells in tumors after 7 days Myc activation (7d tam, red squares) then following 3 days Myc de-activation in IgG control-treated mice (black circles) versus mice treated with αCD4/αCD8 antibody (black squares). Bottom panel shows representative immunohistology. Arrows depict CD3^+^ T cells. T = tumor. Right: Quantification of immunostaining for NKp46^+^ NK cells in tumors after 7 days Myc activation (7d tam), then following 3 days Myc de-activation in IgG control-treated mice versus mice treated with α-asialo-GM1. Bottom panel shows representative examples of the immunohistology. A = airway. T = tumor. (C) (Left) Quantification and representative immunostaining for tumor cell death (TUNEL) following Myc de-activation for 3 days in tumors in CD4^+^/8^+^ T cell competent (IgG-control) versus CD4^+^/8^+^ T cell-deficient mice (αCD4/αCD8). Right: Quantification and representative immunostaining for cell death (TUNEL) following Myc de-activation for 3 days in NKp46^+^ NK cell-competent (IgG-control) versus NKp46^+^ NK cell-deficient mice (α-asialo-GM1 treated). Quantification graphs: FoV = field of view. Small symbols = individual tumors, large symbols = average per mouse. (A) n = 4 (IgG) or 5 (αCD4/αCD8 or α-asialo-GM1). (B) n = 20 or 25 individual tumors from 4 (7d Tam, IgG) or 5 (αCD4/αCD8) mice per time point, respectively. (C) n = 5 mice per treatment group. n = 30 individual tumors from 5 mice per treatment group. Error bars represent the median with interquartile range (CD3, TUNEL) or mean ± SD (NKp46). p values are based on Student’s t test (CD3, TUNEL) or two-way ANOVA (NKp46). NS = non-significant. ^∗^p < 0.05, ^∗∗^p < 0.01, ^∗∗∗^p < 0.001, ^∗∗∗∗^p < 0.0001. See also Figures S1, S6, and S7.

**Figure S6:**
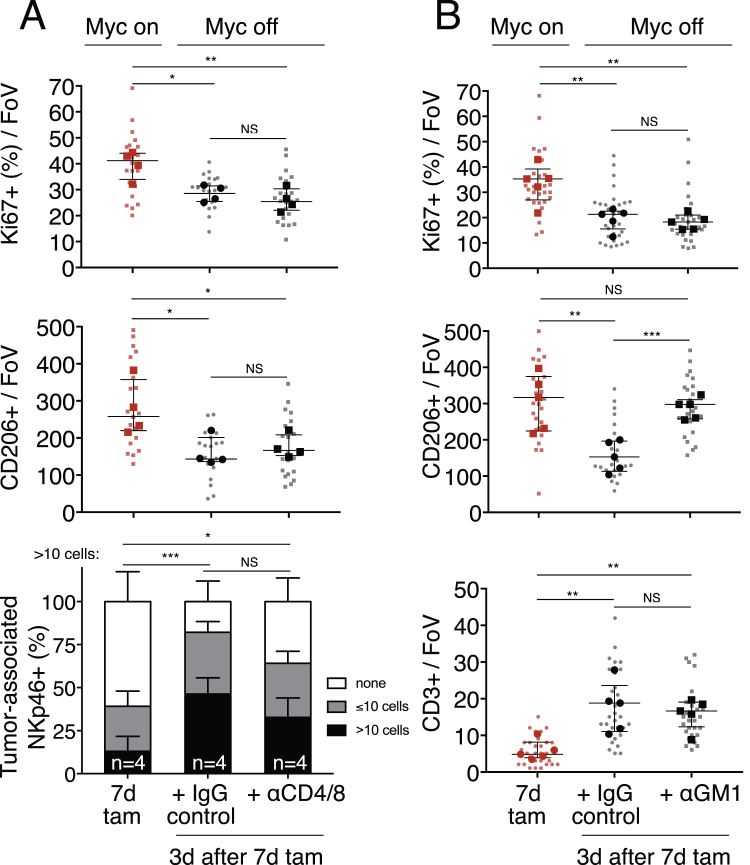
Depletion of NKp46^+^ Cells, but Not of CD4^+^ and CD8^+^ T Cells, Retards Reversion of Myc-Driven Tumor Stromal Changes following Myc De-activation, Related to Figure 7 (A) Quantification of immunostaining for proliferation (Ki67), CD206^+^ macrophages and NK cells (NKp46) following Myc de-activation for 3 days in *KM* tumors of either CD4^+^/CD8^+^ T cell competent animals (IgG-control) or CD4^+^/8^+^ T cell-deficient (αCD4/αCD8-treated) mice compared to 7 days of Myc activation (7d tam). No significant differences in IgG control versus T cell depleted animals are evident. (B) Quantification of immunostaining for proliferation (Ki67), CD206^+^ macrophages and T cells (CD3) following Myc de-activation for 3 days in *KM* tumors of either NKp46^+^ NK cell competent animals (IgG-control) or NKp46^+^ NK cell-deficient (α-asialoGM1-treated) mice. No significant differences in IgG control versus NK-cell depleted animals are evident for proliferation (Ki67) or T cells (CD3). In contrast, NK-cell depleted animals show reduced CD206 efflux following Myc de-activation. Quantification graphs: FoV = Field of View. (A): n = 20-25 individual tumors (small symbols) from 4 (7d Tam, IgG, large symbols) or 5 (αCD4/αCD8) mice per treatment group. (B): n = 25-30 individual tumors (small symbols) from 5 mice (large symbols) per treatment group. Error bars represent the median with interquartile range (Ki67, CD206, CD3) or mean ± SD (NKp46). P values are based on Student’s t test (Ki67, CD206, CD3) or two-way ANOVA (NKp46). NS = non-significant, ^∗^p < 0.05, ^∗∗^p < 0.01, ^∗∗∗^p < 0.001.

The tight temporal coincidence between tumor cell apoptosis and re-entry of T and NK cells (Figure 6B) prompted us to address whether T or NK cells play a causal role in Myc-de-activation-induced tumor regression. To investigate this, we depleted *KM* mice of either CD4^+^ helper and CD8^+^ effector T cells or NK cells using appropriate antibodies (Figure S1I). Systemic anti-CD4/CD8 treatment efficiently ablated all detectable circulating and splenic T cells (Figure 7A) and completely abrogated the re-population of tumors by CD3^+^ T cells following Myc de-activation (Figure 7B). However, absence of CD4^+^/CD8^+^ T cells had no measurable inhibitory effect on induction of tumor apoptosis (Figure 7C), nor did it inhibit macrophage efflux or influx of NK cells (Figure S6A). By contrast, systemic depletion of NKp46^+^ NK cells using asiolo-GM1 antibody (Figures 7A and 7B), while having no impact on Myc deactivation-induced T cell influx (Figure S6B), profoundly inhibited both efflux of macrophages (Figure S6B) and induction of the apoptosis responsible for tumor regression (Figure 7C).

## Discussion

To address the mechanism and dynamics of cooperation between oncogenic KRas^*G12D*^ and Myc in discrete tissues *in vivo*, we used *KM* mice that combine the well-characterized *LSL-KRas*^*G12D*^ mouse lung tumor model (Jackson et al., 2001) with our *Rosa26-LSL-MycER*^*T2*^ mouse (Murphy et al., 2008). Inhalation by *KM* mice of AdV-Cre sporadically co-induces expression of both oncogenic KRas^*G12D*^ and tamoxifen-switchable MycER^T2^ in bronchioalveolar epithelium; in the resulting adenomas, oncogenic KRas^*G12D*^ activity is expressed constitutively while Myc activity may be reversibly superimposed at will. Adeno-Cre activation of KRas^*G12D*^ alone initiates formation of sporadic dysplasias that slowly progress to indolent adenomas and only rarely and sporadically evolve into aggressive adenocarcinomas. Activation of Myc alone had no observable effect on lung tissues. By contrast, sustained co-activation of Myc drove dramatic progression into highly proliferative, invasive, angiogenic, inflammatory, and lethal adenocarcinomas.

Long-term Myc activation studies provide little mechanistic cause-and-effect information as to how Myc deregulation drives progression of indolent KRas^*G12D*^-driven adenomas into aggressive adenocarcinomas. In particular, whether the profound stromal changes that Myc elicits are direct consequences of Myc programming or chronic host tissue responses to intrusion by the tumor is unclear. To distinguish between these two possibilities, we used the remarkably synchronous and rapid *in vivo* Myc activation afforded by the MycER^T2^ switchable system (Christophorou et al., 2005, Littlewood et al., 1995, Pelengaris et al., 2002, Shchors et al., 2006, Wilson et al., 2014) to delineate the temporal sequence of events by which Myc activation drives transition to adenocarcinoma.

Acute MycER^T2^ activation in indolent KRas^*G12D*^-driven lung adenomas rapidly reprogrammed both hematopoietic and endothelial stromal compartments, driving rapid influx of CD206^+^ macrophages prototypically associated with tissue remodeling and immune regulation in diverse tissues (Martinez and Gordon, 2014, Rőszer, 2015) including lung (Aggarwal et al., 2014); rapid loss of T, B, and NK cells; and vasculature remodeling coincident with transition from localized hypoxia to normoxia. These diverse stromal changes preceded any significant expansion of the tumor masses. Hence, the signature “stromatype” of the aggressive adenocarcinomas induced by sustained co-activity of KRas^*G12D*^ and Myc is an immediate, instructed consequence of Myc activation, not an indirect or generic tissue reaction to disruption by the expanding tumor in its midst.

Since MycER^T2^ is activated only in the epithelial compartment of *KM* adenomas, the consequent stromal changes must be mediated by signals originating in that epithelium. A broad search identified only two candidates, CCL9 and IL-23, as induced in lung adenomas with sufficient rapidity (by 8 hr) to be proximal causes of the Myc-induced stromal changes we observe. Both immunohistochemically localized to the TTF1^+^ epithelial compartment. CCL9, a.k.a. MIP-1γ, is a murine ligand for the CCR1/CD191 receptor present on monocytes, T cells, and some immature CD34^+^ myeloid-derived suppressor cells (MDSCs). It is a potent chemo-attractant for monocytes, macrophages, and MDSCs (White et al., 2013), while CCR1 ligation is implicated in progression of adenomas to carcinomas (Kitamura et al., 2007, Koizumi et al., 2007) and inflammatory lung pathologies (Kitamura et al., 2015). IL-23 is a pro-inflammatory cytokine that suppresses wound resolution and is pro-tumorigenic in many tissues (Langowski et al., 2006), including lung (Baird et al., 2013). IL-23 is the principal trigger of T_H_17 lymphocytes, an ROR1γ-dependent T cell subset whose consequent secretion of IL-17 and IL-22 induces, in turn, production of diverse inflammatory cytokines, chemokines, and prostaglandins by many stromal cell types (Gaffen et al., 2014). T_H_17 cells are also highly immunosuppressive (Bailey et al., 2014, Chang, 2015, Chang et al., 2014). IL-23 is also a potent suppressor of innate immunity, most notably NK cells (Teng et al., 2010). IL-23 shares a common p40 subunit with IL-12 that, in many ways, antagonizes IL-23 in cancers by boosting anti-tumor immunity (Ngiow et al., 2013, Teng et al., 2012) and promoting injury resolution and subsequent tissue re-normalization (Ngiow et al., 2013).

**Figure S7:**
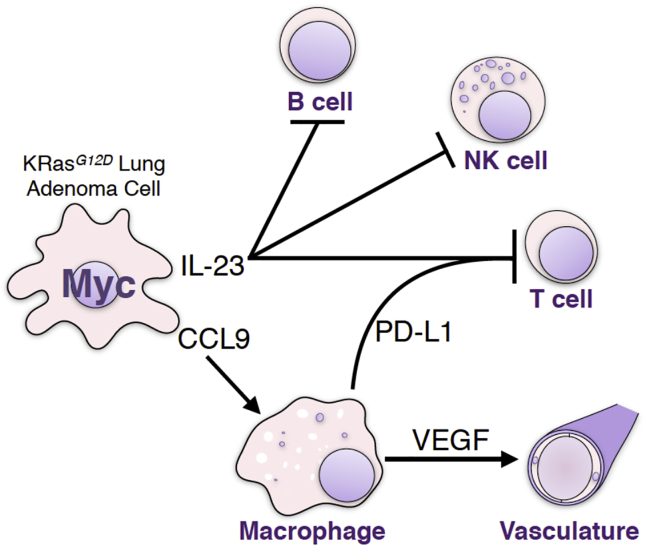
Myc Instructs Stromal Changes in Lung Adenomas, Related to Figures 2, 3, 4, and 7 Schematic representation of Myc-induced stromal changes in lung adenocarcinoma. Activation of deregulated Myc in epithelial KRas^*G12D*^-driven lung adenoma cells rapidly leads to efflux of B, T and NK lymphocytes and recruitment of macrophages. Myc-induced IL-23 promotes efflux of B, T and NK cells whereas Myc-dependent CCL9 recruits macrophages. The macrophages stimulate angiogenesis via overt VEGF production and repel T cells via surface expression of PD-L1. Myc-dependent tumor progression requires IL-23 and CCL9 signaling to NK cells and macrophages, respectively. De-activation of deregulated Myc in established KRas^*G12D*^-Myc adenocarcinomas leads to the rapid reversal of these stromal changes, tumor cell death and regression.

Co-blockade of both CCL9 and IL-23 profoundly inhibited all the diverse stromal changes that Myc elicits, suppressed Myc-induced tumor cell proliferation, and triggered abrupt onset of apoptosis. Blockade of CCL9 alone had little impact on tumor cell proliferation or apoptosis but completely inhibited Myc-induced macrophage influx, angiogenesis, and T cell loss. It also reduced loss of B cells yet had no inhibitory impact on Myc-driven loss of juxta-tumoral NK cells. By contrast, blockade of IL-23 alone had no inhibitory impact on macrophage influx or angiogenesis but strongly suppressed T cell loss, completely blocked the loss of NK and B cells, mildly suppressed tumor cell proliferation, and unlike blockade of CCL9 alone, triggered widespread tumor cell apoptosis. From these studies, we draw several conclusions (Figure S7). First, CCL9 and IL-23 are the principal epithelial signals that instruct the immediate and diverse stromal changes that Myc elicits. Second, while both CCL9 and IL-23 are crucial for the rapid loss of T and B cells following Myc activation, CCL9 alone is required to drive macrophage recruitment and angiogenesis, while IL-23 alone is required for rapid exclusion of NK cells. Third, since blockade of IL-23, but not CCL9, triggers substantial tumor cell apoptosis, IL-23 plays a unique role in survival and maintenance of Myc-driven lung tumors.

Myc activation rapidly induced a profound angiogenic switch, evident by blood vessel permeability and loss of integrity that coincided with VEGF activity. VEGF was potently induced in the macrophage compartment following Myc activation, implicating macrophages as the principal angiogenic mediators, consistent with the inhibition of Myc-induced angiogenesis by CCL9 blockade and the widely reported angiogenic role of macrophages (Chanmee et al., 2014, Riabov et al., 2014) (Figure S7). In exploring the mechanism underlying the peculiar obligate role of CCL9 in Myc-driven exclusion of T and B cells, but not NK cells, we noted the rapid rise in the lung tumors of cells expressing the immunosuppressive ligand PD-L1 (a.k.a. B7-H1 or CD274) following Myc activation. However, PD-L1 expression was not directly induced by Myc, as recently reported (Casey et al., 2016), but entirely confined to incoming F4/80^+^ macrophage-like cells. These are known to express PD-L1 (Igarashi et al., 2016), whose increase is a simple consequence of CCL9-dependent macrophage influx. Systemic blockade of PD-L1 completely abrogated Myc-induced loss of CD3^+^ T cells—confirming macrophage-derived PD-L1 as necessary for Myc-induced T cell depletion—but had no effect on NK exclusion or macrophage influx. The extremely rapid Myc-induced, CCL9-dependent T cell depletion occurred without detectable T cell apoptosis, and this, together with the equally rapid re-appearance of T cells in adenomas upon Myc de-activation, suggests that migration of T cells out and in to adenoma tissue is the likely mechanism for the rapid T cell dynamics that Myc elicits.

We next assessed Myc’s role in maintaining lung adenocarcinomas. Acute Myc de-activation in established *KM* adenocarcinomas triggered rapid and synchronous regression of all tumors, characterized by a direct reversal of all Myc-induced stromal changes: macrophages rapidly exited the tumor masses; tumor vasculature reformed into discrete vessels (“vascular re-normalization”) and widespread hypoxia reappeared; T, B, and NK cells rapidly repopulated the tumors and their environs; and tumor cell apoptosis and tumor regression rapidly ensued. Remarkably, even very short-term (7 days) activation of ectopic Myc was sufficient to establish such Myc dependency in tumors. Acquired dependency on driver oncogenes (oncogene addiction) is a pervasive phenomenon that provides the underpinning rationale for most current targeted therapies. Its mechanism remains unclear, although co-dependency of oncogenic mutations, the abrupt re-instatement of cell intrinsic or immune checkpoints, asynchrony in attenuation rates of mitogenic versus survival signals, and collapse of tumor stroma have all been implicated (Restifo, 2010, Sharma et al., 2006, Sodir et al., 2011, Tran et al., 2011). Intriguingly, once the Myc-driven *KM* tumors had regressed back to their previous KRas^*G12D*^-only adenoma state and size, tumor regression stalled indefinitely. Similarly incomplete regression upon Myc de-activation has been seen in other Myc transgenic models (Boxer et al., 2004, Podsypanina et al., 2008, Tran et al., 2008). It contrasts with the complete regression—indeed, eradication—seen upon inhibition of Myc in KRas^*G12D*^-driven lung tumors via the dominant negative Omomyc mutant (Soucek et al., 2008, Soucek et al., 2013)—an apparent discrepancy that likely arises because Omomyc inhibits not only *Myc* transgenes, but also endogenous Myc. The rapidity with which Myc dependency arises is incompatible with the idea that Myc addiction reflects acquired co-dependency upon other oncogenic mutations. Rather, our data favor a tight temporal and spatial co-dependence between Myc driven tumor cell expansion and Myc-driven tumor stroma. However, precisely which attributes of the Myc-instructed tumor stroma are so essential for tumor maintenance are less clear. T cells do not appear to be involved, since abrogation of Myc-driven T cell expulsion by PD-L1 blockade has no inhibitory impact on Myc-driven lung tumor growth, progression, or burden. Moreover, while systemic depletion of CD4^+^ and CD8^+^ T cells prevented T cell re-colonization of tumors following Myc de-activation, it does not measurably retard either stromal collapse or tumor regression. By contrast, our data do suggest a possible role for NK cells in limiting *KM* tumor growth. Myc rapidly induced expression of Rae-1, the principal activating ligands for the NK cell NKG2D receptor, in adenoma epithelial cells. It also downregulated MHC class I, a potent trigger of NK cell activation as well as a means of adaptive T cell evasion (Töpfer et al., 2011). Given two such potent NK cell activators, it is difficult to see how *KM* tumors could survive without the rapid clearance of NK cells that IL-23 mediates. Indeed, blockade of IL-23 in Myc-stimulated *KM* tumors triggered profound tumor apoptosis that was not seen when expulsion of T cells alone was blocked by PD-L1 blockade. We also note that the transient wave of apoptosis (Figure 6C) responsible for regression of *KM* tumors induced by Myc deactivation precisely coincides temporally with transient influx of NK cells and is significantly suppressed when NK cells, but not CD4^+^CD8^+^ T cells, are systemically ablated.

Advantageous though it may be for tumors, the question remains as to why Myc is bestowed with the capacity to drive rapid clearance of adaptive and innate lymphocytes cells, a property of Myc we see across diverse tissues. We guess the answer lies in the physiological role of Myc as the transcriptional coordinator of the diverse array of intra- and extracellular processes needed for regeneration in lung (Dong et al., 2011) and other tissues after injury. Such regeneration requires complex, tissue-specific interplay between epithelial, myeloid, mesenchymal, and endothelial stromal elements needed to clear debris, seal the wound, and regenerate the tissue. In sterile situations (where pathogens are no complication), this rapid outgrowth phase is typically associated with rapid influx of immunosuppressive monocytic and myeloid-derived suppressor cells (Wynn and Vannella, 2016) followed by a resolution phase marked by return of lymphoid cells (D’Alessio et al., 2009, Wu et al., 2013) during which epithelial regeneration attenuates and extensive tissue and vascular remodeling reconstruct the original tissue architecture. The similarities between this biphasic physiological process, regeneration followed by resolution, and Myc-driven tumorigenesis followed by Myc de-activation-induced tumor regression, are provocative.

## STAR★Methods

### Key Resources Table

**Table:** 

REAGENT or RESOURCE	SOURCE	IDENTIFIER
**Antibodies**		

Rat monoclonal anti-CD45 [30-F11]	BD Biosciences	Cat#: 553076, RRID:AB_394606
Rat monoclonal anti-F4/80 [Cl:A3-1]	Bio-Rad	Cat#: MCA497R, RRID:AB_323279
Goat polyclonal anti-MMR/CD206	R&D Systems	Cat#: AF2535, RRID:AB_2063012
Rabbit monoclonal anti-CD3 [SP7]	ThermoFisher	Cat#: RM-9107-RQ
Rabbit polyclonal anti-CD274/PD-L1	Abcam	Cat#: ab58810, RRID:AB_940872
Rabbit monoclonal anti-Ki67 [SP6]	ThermoFisher	Cat#: RM-9106-S1, RRID:AB_149792
Rat anti-CD31/PECAM1 [MEC13.3]	BD Biosciences	Cat#: 550274, RRID:AB_393571
Rat monoclonal anti-Pan-Endothelial cell antigen/PVLAP [MECA32]	BD Biosciences	Cat#: 550563, RRID:AB_393754
Mouse monoclonal anti-VEGF:VEGFR2 (GV39M)	EastCoast Bio	Cat#: CD301
Rabbit monoclonal anti-IL23p19	Abcam	Cat#: ab45420, RRID:AB_2124515
Goat polyclonal anti-CCL9/10/MIP1γ	R&D Systems	Cat#: AF-463, RRID:AB_2071817
Rabbit monoclonal anti-TTF1 [EP1584Y]	Abcam	Cat#: ab76013, RRID:AB_1310784
Mouse monoclonal anti-TTF1 [8G7G3/1]	Abcam	Cat#: ab72876, RRID:AB_1271363
Mouse monoclonal anti-Hypoxyprobe [4.3.11.3]	Hypoxyprobe	Cat#: HP1-100
Polyclonal goat anti-Rae-1 Pan-specific	R&D Systems	Cat#: AF1136, RRID:AB_2238016
Rat monoclonal anti-MHC1 [ER-HR52]	Novus Biologicals	Cat#: NB100-64952, RRID:AB_964497
Rat monoclonal anti-CD45R/B220 [RA3-6B2]	ThermoFisher	Cat#: MA1-70098, RRID:AB_1072441
Rat anti-CD335/NKp46 [29A1.4]	Biolegend	Cat#: 137601, RRID:AB_10551441
Fluorescein-labeled Lycopersicon Esculentum (Tomato) Lectin	Vector Labs	Cat#: FL-1171, RRID:AB_2307440
Rhodamine-labeled Ricinus communis Agglutinin I	Vector Labs	Cat#: RL-1082, RRID:AB_2336710
LEAF-purified Rat anti-mouse CD274/PD-L1 [10F.9G2]	Biolegend	Cat#: 124309, RRID:AB_2291192
LEAF-purified Rat IgG2b, κ Isotype control [RTK4530]	Biolegend	Cat#: 400637, RRID:AB_2086803
LEAF-purified Mouse anti-mouse IL23p19 [MMp19B2]	Biolegend	Cat#: 513806, RRID:AB_2562073
LEAF-purified Mouse IgG2b, κ Isotype control [MPC-11]	Biolegend	Cat#: 400339
Goat polyclonal anti-mouse CCL9/10/MIP1γ	R&D Systems	Cat#: AF463, RRID:AB_2071817
Goat IgG control	R&D Systems	Cat#: AB-108-C, RRID:AB_354267
Rat anti-mouse CD4 [GK1.5]	BioXcell	Cat#: BE0003, RRID:AB_1107636
Rat anti-mouse CD8 [2.43]	BioXcell	Cat#: BE0061, RRID:AB_1125541
Rat IgG2b control [LTF2]	BioXcell	Cat#: BE0090, RRID:AB_1107780
Rabbit anti-mouse asialo-GM1	Wako Chemicals	Cat#: 986-10001, RRID:AB_516844
Rabbit IgG control	R&D Systems	Cat#: AB-105-C, RRID:AB_354266
Rat anti-mouse CD3ε [145-2C11]	eBioscience /ThermoFisher	Cat#: 17-0031, RRID:AB_469313
Rat anti-mouse CD8α [53-6.7]	eBioscience /ThermoFisher	Cat#: 12-0081, RRID:AB_465529
Rat anti-mouse CD4 [RM4-5]	eBioscience /ThermoFisher	Cat#: 14-0042, RRID:AB_467066
Rat anti-mouse CD4 [GK1.5]	eBioscience /ThermoFisher	Cat#: 11-0041, RRID:AB_464891
Rat anti-mouse CD45 [30-F11]	eBioscience /ThermoFisher	Cat#: 14-0451, RRID:AB_467252
Rat anti-mouse CD335/NKp46 [29A1.4]	eBioscience /ThermoFisher	Cat#: 11-3351, RRID:AB_1210843

**Chemicals**		

Tamoxifen	Sigma-Aldrich	Cat#: T5648
7-AAD Viability staining solution	eBioscience	Cat#: 00-6993-50

**Critical Commercial Assays, Macrophage Isolation**		

Mouse Inflammation Antibody Array - Membrane	Abcam	Cat#: ab133999
ApopTag Fluorescein Direct *In Situ* Apoptosis Detection Kit (TUNEL)	Millipore	Cat#: S7160
High Capacity cDNA RT kit	Applied Biosystems	Cat#: 4374966
Fast Sybr Green	Applied Biosystems	Cat#: 4385612
RNeasy Plus Mini Kit	QIAGEN	Cat#: 74134
Anti-F4/80-biotin [REA126]	Miltenyi Biotech	Cat#: 130-101-893, RRID: AB_2651713
Anti-F4/80 Microbeads, Ultra Pure	Miltenyi Biotech	Cat#: 130-110-443
RNAscope 2.5 HD Reagent Kit-BROWN	Advanced Cell Diagnostics	Cat#: 322300
TSA Plus multi-fluorophore detection kit	PerkinElmer	Cat#: NEL760001KT

**Experimental Models: Organisms/Strains**		

*LSL-KRas*^*G12D*^	Jackson et al., 2001	N/A
*Rosa26LSLMyc*^*ERT2*^	Murphy et al., 2008	N/A

**Software and Algorithms**		

FlowJo (version X)	FlowJo	RRID: SCR_008520; https://www.flowjo.com/
ImageJ/Fiji	Open source	RRID: SCR_003070
Graphpad Prism	GraphPad Software	RRID: SCR_002798; https://www.graphpad.com/scientific-software/prism/

### Contact for Reagent and Resource Sharing

Further information and requests for resources and reagents should be directed to and will be fulfilled by the Lead Contact, Gerard I. Evan (gie20@cam.ac.uk).

### Experimental Model and Subject Details

#### Mice and in vivo procedures

All treatments and procedures of mice were conducted in accordance with protocols approved by the Institutional Animal Care and Use Committee at UCSF and Home Office UK guidelines under project licenses to G.I.E., (70/7586, 80/2396) at the University of Cambridge. Mice were maintained on regular diet in a pathogen-free facility on a 12 hr light/dark cycle with continuous access to food and water. *Lsl-Kras*^*G12D*^ (Jackson et al., 2001) and *LSL-Rosa26*^*MIE/MIE*^ (Murphy et al., 2008) mice have been described previously. For activation of MycER^T2^, Tamoxifen (Sigma; T5648) dissolved in peanut oil was administered daily by intraperitoneal (IP) injection for a maximum of 6 weeks at a dose of 1 mg/20 g body mass. To deliver adenovirus-Cre recombinase (AdV-Cre), mice were anesthetized with 2.5% Avertin (250 ml/20 g body mass) or isoflurane (Zoetis, IsoFlo 250 ml) and 5x10^7^ plaque-forming units of AdV-Cre were administered as described previously (Fasbender et al., 1998). Mice were between 8 and 12 weeks old at time of AdV-Cre infection, and between 20-28 weeks of age at time of euthanasia and analysis. Equivalent female and male age-matched littermates were divided over experimental and control mice. Lectins were administered by retro-orbital injection prior to sacrifice and Hypoxyprobe and blocking antibodies for PD-L1, IL23p19, CCL9, CD4, CD8 and asialo-GM1 were administered by IP injection at doses and frequency described in method details and Figure S1.

### Method Details

#### Immunohistochemistry and Immunofluorescence

Mice were euthanized and cardiac-perfused with PBS followed by 10% neutral-buffered formalin (Sigma-Aldrich, 501320). Lungs were removed, fixed overnight in neutral-buffered formalin and processed for paraffin embedding. Tissue sections were stained with hematoxylin and eosin (H&E) using standard reagents and protocols. For frozen sections, lungs were directly embedded in OCT (VWR Chemicals, 361603E) and either frozen on dry ice or snap frozen in liquid Nitrogen, embedded in OCT, and stored at −80°C. For immunofluorescence analysis of VEGF bound to its receptor (VEGF:VEGFR2), OCT frozen cryostat-cut 10 μm sections were air-dried, serum blocked and incubated with the GV39M antibody overnight. For immunohistochemical and immunofluorescence analysis, sections were de-paraffinized, rehydrated, and boiled in a microwave for 10 minutes in 10 mM citrate buffer (pH 6.0) or treated with 20 μg/ml Proteinase K for antigen retrieval. Antibodies were incubated overnight at 4°C except for anti-CD3, which was incubated for 20 minutes at room temperature. Antibodies used (see key resources table): F4/80 (Cl:A3-1); MMR/CD206; CD45 (30-F11); CD3 (SP7); CD274/PD-L1; Ki67 (SP6); c-Myc (Y69); CD31/PECAM (MEC13.3); Pan-endothelial cell antigen/PVLAP (MECA32); IL23p19; CCL9/10/MIP1γ; TTF1 (EP1584Y); TTF1 (8G7G3/1); Hypoxyprobe (4.3.11.3); Pan-Rae-1; MHC1 (ER-HR52); CD45R/B220 (RA3-6B2); CD335/NKp46 (29A1.4). Commercial antibodies were purchased from Cedarlane, Merck, Bio-Rad, R&D Systems, BD, ThermoFisher, Abcam, East Coast Biologics, Novus, Hypoxyprobe and BioLegend. HRP- conjugated secondary antibodies (Vectastain Elite ABC Kits: PK-6200; Universal, PK-6101; Rabbit, PK-6104; Rat, PK-6105; Goat) were applied for 30 min and visualized with DAB (Vector Laboratories; SK-4100), or secondary Alexa Fluor 488 or −455 dye-conjugated antibodies (Life Technologies) applied for 30 minutes at room temperature. Fluorescence antibody-labeled slides were mounted in fluorescent mounting medium (Prolong Molecular Probes; P36934) post-treatment with 0.5 μg/ml Hoechst counter-stain. TUNEL analysis was performed using the Apoptag Fluorescein *In Situ* Apoptosis Detection Kit (Millipore; S7110) according to manufacturer’s instructions. Briefly, tissue sections were pre-treated in Proteinase K (20 μg/ml) for 15 min at room temperature, washed in deionized water twice for 2 min each, and returned to PBS. Sections were then covered with equilibration buffer for a minimum of 2 min followed by incubation at 37°C for 1 hr with a 1:5 dilution of TdT enzyme in reaction buffer, followed by fluorescein anti-digoxigenin conjugated secondary antibody, washed, and mounted. To stain tissues systemically for hypoxia, 60 mg/kg hypoxyprobe-1 (1-{[2-hydroxy-3-piperdinyl] propyl}-2-nitroimidazole hydrochloride) (Hypoxyprobe; HP1-100 kit) was administered through IP injection in saline 15 min prior to euthanasia. Protein adducts of reductively activated pimonidazole were identified through immunohistochemistry in fixed tissues with a monoclonal antibody against hypoxyprobe-1.

RNA-ISH for *MycER*^*T2*^ was performed with a custom-designed probe targeting nucleotides 2110-3619 of Myc-ER-ires-EGFP (Murphy et al., 2008), amplified with RNAscope 2.5 HD Reagent Kit (Advanced Cell Diagnostics; 322300) and developed with the TSA Plus kit (PerkinElmer; NEL760001KT) according to manufacturer’s instructions. Images were collected with an Axiovert 5100 TV inverted fluorescence microscope (Zeiss) and Open Lab 3.5.1 software, or with an Axiovert 100 inverted microscope (Zeiss) equipped with a Hamamatsu Orca digital camera (University of California, San Francisco), and a Zeiss Axio Imager M2 microscope and Axiovision Rel 4.8 software (University of Cambridge).

#### Isolation of mouse lung tumor-associated macrophage RNA

After heart-perfusion with PBS, mouse lungs were harvested and macrophages isolated following the MACS cell-separation protocol according to manufacturer’s instructions (Miltenyi Biotec). Briefly: lung tissue was minced, incubated 15 minutes at 37°C in Ca^2+^/Mg^2+^-free PBS with 0.5% EDTA, passed through 40 μm Falcon cell strainers (352340) and re-suspended in erythrocyte lysis buffer (RBC Tris-buffered ammonium chloride pH7.2) followed by dissociation buffer (PBS pH7.2 with 2mM EDTA). The resulting homogenate was incubated with anti-F4/80 biotin-conjugated antibody (Miltenyi Biotec, 130-101-893) followed by streptavidin-conjugated microbeads (Miltenyi Biotec, 130-101-893), or incubated with anti-F4/80 microbead-conjugated antibody (Miltenyi Biotec, 130-110-443), passed through magnetic columns and the macrophages then transferred to TRizol (Ambion, 15596018) for RNA isolation with the RNeasy Plus Mini Kit (QIAGEN, 74134), according to manufacturer’s instructions.

#### Immune Array and determination of MycER^T2^, PD-L1, and VEGFA mRNA

14 weeks post AdV-Cre *KM* mouse lungs were isolated 8 hours after IP injection with either oil or tamoxifen and then snap frozen in liquid nitrogen. Whole (tumor-laden) lung protein samples were isolated and incubated on a mouse inflammation antibody array (Abcam, ab133999) according to manufacturer instructions. The intensity of the signals was analyzed using ImageJ.

Total RNA (0.5-1 mg) isolated from F4/80^-^ lung- and tumor tissue or F4/80^+^ macrophages was reverse transcribed using the High Capacity cDNA RT kit (Applied Biosystems, 4374966). Real-time quantitative RT–PCR (Fast Sybr Green, Applied Biosystems, 4385612) was used to quantify mRNA levels. The *TBP* and *β-actin* genes were used as an internal amplification control. TBP forward primer: 5′-ACTTCGTGCAAGAAATGCTGAAT-3′, TBP reverse primer: 5′- CAGTTGTCCGTGGCTCTCTTATT-3′. β-actin forward primer: 5′-GACGATATCGCTGCGCTGG −3′, β-actin reverse primer: 5′-CCACGATGGAGGGGAATA-3′. MycER^T2^ primers; forward: 5′-ATTTCTGAAGACTTGTTGCGGAAA-3′, reverse: 5′- GCTGTTCTTAGAGCGTTTGATCATGA-3′ (Murphy et al., Cancer Cell 14 (6), 2008). PD-L1 primers; forward: 5′-GACCAGCTTTTGAAGGGAAATG-3′, reverse: 5′-CTGGTTGATTTTGCGGTATGG-3′. VEGFA primers were from Bio-Rad (10025636, qMmuCED0040260). Real-time PCR reactions were performed on an Eppendorf Mastercycler Realpex 2 and analyzed with accompanying software.

#### Blocking antibodies and Flow Cytometric analysis

For antibody blocking experiments, mice were IP injected with either anti-IL23p19 or anti-CCL9 individually or together (see Figures 3 and S4) or with anti-PD-L1 every two days, starting one day before Myc activation by tamoxifen injection (see Figures 4 and S1). For tumor regression studies, antibody ablation of CD4^+^ and CD8^+^ T cells or NKp46^+^ cells was initiated four days before tamoxifen withdrawal (see Figures 7, S1, and S6). Concentrations of antibodies and their isotype controls were adjusted in PBS as follows (see key resources table): IL23p19 (MMp19B2) and Mouse IgG2b κ Isotype control (MPC-11) 150 μg/mouse/IP; CCL9/10/MIP1γ (AF463) and goat IgG control (AB-108-C) 50 μg/mouse/IP; CD274/PD-L1 (10F.9G2) and Rat IgG2b κ Isotype control (RTK4530) 150 μg/mouse/IP; CD4 (GK1.5) and CD8 (2.43) and Rat IgG control (LTF2) 200 μg/mouse/IP; asialo-GM1 and Rabbit IgG control 100 μl/mouse/IP and 100 μg/mouse/IP, respectively. For Flow Cytometric identification of CD4 and CD8 positive T cells in spleen and blood of anti-CD4 and anti-CD8 treated mice, CD3ε (145-2C11), CD8α (53-6.7), CD4 (RM4-5, GK1.5) and CD45 (30-F11)-specific antibodies were used. For Flow Cytometric identification of NKp46 positive NK cells in blood and spleen of anti-asialo-GM1 treated mice NKp46 (29A1.4)-specific antibody was used. For isolation and analysis of lung-specific NKp46^+^ cells whole lung tissue was minced, incubated 15 minutes at 37°C in Ca^2+^/Mg^2+^-free PBS with 0.5% EDTA, passed through 40 μm Falcon cell strainers (352340) and re-suspended in erythrocyte lysis buffer (RBC Tris-buffered ammonium chloride pH7.2) followed by dissociation buffer (PBS pH7.2 with 2mM EDTA). The cell suspension was incubated with FITC-NKp46-specific antibody (eBioscience, 11-3351). Flow Cytometry was performed on an Accuri C6 Flow cytometer (BD Biosciences) with accompanying software. Commercial antibodies were purchased from R&D Systems, BioLegend, BioXcell and Wako Chemicals.

#### Lectins

Lectin dyes Fluorescein-*Lycopersicon esculentum* (FL-1171) and Rhodamine-*Ricinus communis* agglutinin I (RL-1082) were obtained from Vector Labs. Three minutes before sacrifice 100 μL of a 1:2 Fluorescein:Rhodamine solution was administered by retro-orbital injection. Lungs were then harvested and fluorescence imaged on de-paraffinized tissue sections counterstained with Hoechst dye.

### Quantification and Statistical Analysis

#### Scanning H&E slides, ImageJ, and GraphPad.

H&E sections were scanned with an Aperio AT2 microscope (Leica Biosystems) at 20X magnification (resolution 0.5 microns per pixel) and analyzed with Aperio Software. Quantifications were performed using ImageJ. Statistical analysis was performed in GraphPad. Data points on the scatter dot graphs that portray quantification per Field of View represent one tumor (small data points) and averages per mouse (large data points) simultaneously. A minimum of five tumors per mouse were analyzed. For comparison, quantification of histological markers was only performed on tumor sections stained at the same time. For endothelial cell marker CD31 the immunohistochemistry staining intensity was calculated as percentage CD31-positive area per Field of View using ImageJ. For proliferation marker Ki67 the immunohistochemistry staining was quantified as percentage of total cells per Field of View using ImageJ. For quantification of NK cell marker NKp46 a complete section of the lung of a mouse was analyzed. Scoring (none, ≤ 10 cells, > 10 cells) was based on the amount of juxta-tumoral NKp46^+^ cells visible. Only tumors directly lining and associated with clearly distinguishable vasculature were taken into consideration. P values are derived from mouse-average comparisons between groups and determined via Student’s t test or two-way ANOVA (see figure legends). Bar graphs are represented as mean with standard deviation. Quantifications in scatter dot-plot graphs show median with interquartile range.

## Author Contributions

R.M.K. and G.I.E. conceived the project. R.M.K., N.M.S., L.B.S., T.D.L., and G.I.E. designed experiments. R.M.K. performed all experiments. N.M.S., C.H.W., D.L.B., and L.P. assisted with some experiments. R.M.K., T.D.L., and G.I.E. wrote the manuscript. G.I.E. supervised the study. All authors discussed results and revised the manuscript.
